# Rational design of mesoporous chiral MOFs as reactive pockets in nanochannels for enzyme-free identification of monosaccharide enantiomers†

**DOI:** 10.1039/d2sc05784k

**Published:** 2023-01-17

**Authors:** Junli Guo, Xuao Liu, Junjian Zhao, Huijie Xu, Zhida Gao, Zeng-Qiang Wu, Yan-Yan Song

**Affiliations:** a College of Sciences, Northeastern University Shenyang 110819 China yysong@mail.neu.edu.cn; b School of Public Health, Nantong University Nantong 226019 China zqwu@ntu.edu.cn

## Abstract

Monosaccharides play significant roles in daily metabolism in living organisms. Although various devices have been constructed for monosaccharide identification, most rely on the specificity of the natural enzyme. Herein, inspired by natural ionic channels, an asymmetrical MOF-in-nanochannel architecture is developed to discriminate monosaccharide enantiomers based on cascade reactions by combining oxidase-mimicking and Fenton-like catalysis in homochiral mesoporous CuMOF pockets. The identification performance is remarkably enhanced by the increased oxidase-mimicking activity of Au nanoparticles under a local surface plasmon resonance (LSPR) excitation. The apparent steady-state kinetic parameters and nano-fluidic simulation indicate that the different affinities induced by Au-LSPR excitation and the confinement effect from MOF pockets precipitate the high chiral sensitivity. This study offers a promising strategy for designing an enantiomer discrimination device and helps to gain insight into the origin of stereoselectivity in a natural enzyme.

## Abstract 2

An asymmetrical architecture is developed to discriminate monosaccharide enantiomers based on enzyme-like cascade reactions in homochiral CuMOF pockets. The increased enzyme-like activity under LSPR excitation enhanced the identification performance.

## Graphical abstract

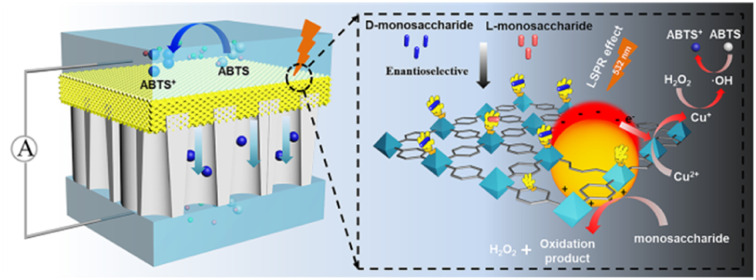

## Introduction

Chirality is essential in different fields, such as living matter, medical sciences, food chemistry, and drug manufacturing.^1^ As a fundamental molecule, chiral monosaccharides play vital roles in the biological activity of all living organisms.^2,3^ Although monosaccharide enantiomers have similar taste quality,^4^l-monosaccharides are non-transportable through living systems. d-Monosaccharides are the natural substrate for daily metabolism.^4^ Therefore, chiral recognition of monosaccharides has always been a hot topic in chemical and biological research. Compared with colorimetric^5^ and fluorescence methods,^6^ nanochannel-based enantiomer recognition techniques have shown great potential in discriminating monosaccharide enantiomers due to their distinct advantages of continuous operation, high sensitivity, and selectivity.^7–9^ To date, many nanochannel-based discrimination systems have efficiently discriminated enantiomers by modifying nanochannels with natural enzymes as chiral receptors.^10,11^ Although these enzyme-modified nanochannels exhibit high selectivity for chiral monosaccharides, their fragile nature and the high cost of natural enzyme application limit their stability and practicability in harsh environments.

Metal–organic frameworks (MOFs) are widely recognized as promising building blocks for constructing catalytic systems due to their microporous structure and having plenty of functional groups.^12^ In the past few years, several MOFs have demonstrated similar performance as biological enzymes.^13–16^ For example, Fe, Cu, Co, Ni, or Ce containing MOFs presented excellent enzyme-mimicking activity.^17^ In addition, due to the intrinsic frame structure and rich surface chemistry, MOFs are easily endowed with chiral properties, becoming one of the most promising hosts for selective adsorption and separation of racemic mixtures.^18^ However, the microporous structure of MOFs^19^ limits access to active sites, thus hindering their practical applications in this field. Many efforts have been made to prepare mesoporous MOFs to improve the accessibility and the catalytic efficiency of MOFs by introducing template molecules, adjusting the length of ligands, and treating MOFs with special solvents.^20–22^ However, due to complex signal transduction in chiral recognition using bulk MOF powders, the application of mesoporous MOFs for enantiomer discrimination has rarely been reported, despite the significant advantages of this approach.

**Scheme 1:**
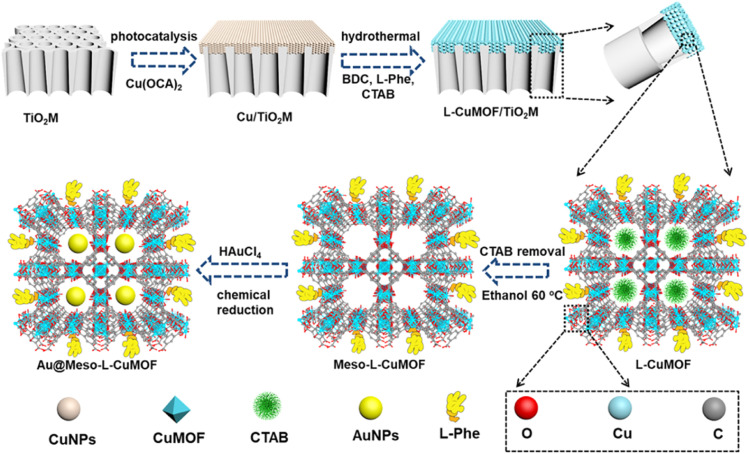
Schematic illustration of preparing Au@Meso-l-CuMOF/TiO_2_M from TiO_2_M.

Inspired by mimicked biological channels, artificial ionic nanochannels have been applied in recent years in different fields, including energy conversion,^23^ biosensing,^24^ and others.^25^ Compared to individual material-based channels, asymmetric artificial nanochannels with different chemical compositions along the channels exhibit remarkably novel functions and properties.^26^ Herein, asymmetrically grown MOF structures in a free-standing anodic TiO_2_ nanochannel membrane (TiO_2_M) featuring excellent photocatalytic activity and chemical stability (Scheme 1) are proposed for highly selective discrimination of monosaccharide enantiomers *via* mesoporous homochiral CuMOF (Meso-l-CuMOF) and artificial oxidase. In particular, an interfacial growth method to achieve asymmetrical MOF decoration by utilizing the catalytic activity of TiO_2_ is reported for the first time. Gold nanoparticles (AuNPs), a kind of artificial oxidase, are filled in Meso-l-CuMOF pockets through an *in situ* reduction approach. This nanochannel-based monosaccharide sensing device eliminates the requirement for natural enzymes and is economical and stable. More importantly, experimental results and numerical simulations indicate that a better-localized surface plasmon resonance (LSPR) mediated ionic product conversion is achieved owing to the nanoconfinement effect of the Meso-l-CuMOF membrane, resulting in a stereoselective and substrate-selective robust sensing device. This study offers inspiration to design and prepare low-cost and stable asymmetrical nanochannels for monosaccharide enantiomer discrimination.

## Results and discussion

**Fig. 1:**
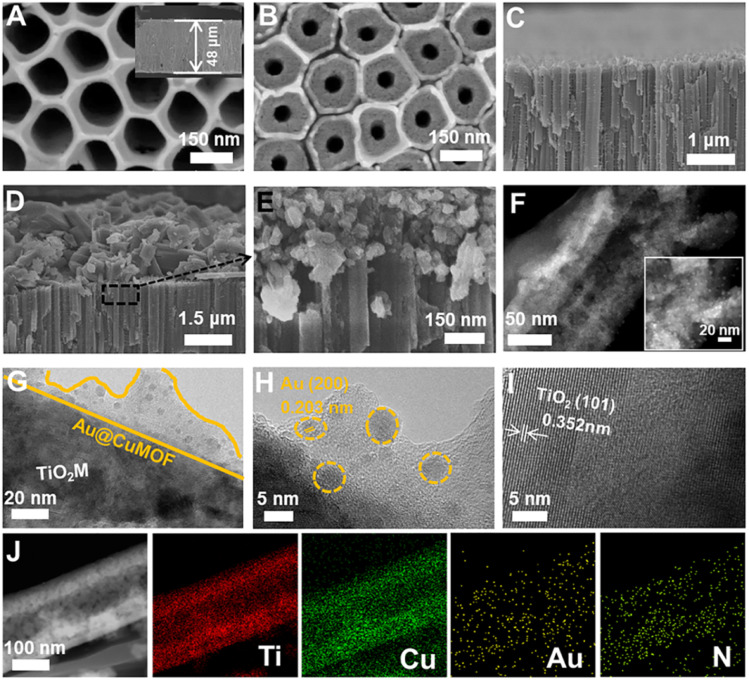
Schematic illustration of preparing Au@Meso-l-CuMOF/TiO_2_M. SEM images: (A) top view, (B) bottom view, and (C) side view of TiO_2_M. (D and E) Side view of Meso-l-CuMOF/TiO_2_M. (F) HAADF-STEM, (G) TEM and (H and I) HRTEM images of Au@Meso-l-CuMOF/TiO_2_M. (J) Elemental mapping of Au@Meso-l-CuMOF/TiO_2_M.

Scheme 1 schematically illustrates the procedure for asymmetrical growth of Meso-l-CuMOF pockets and oxidase-mimicking Au NPs in TiO_2_M. Benefitting from the homochiral environment of Meso-l-CuMOF, the constructed nanochannels show different affinities for glucose enantiomers. This distinct affinity enables one of the enantiomers to be transported through mesoporous MOFs and further oxidized by oxidase-mimicking AuNPs. The generated H_2_O_2_ is *in situ* monitored by the *I*–*V* properties of nanochannels utilizing the Fenton-like activity of a CuMOF. TiO_2_M prepared by electrochemical anodization (details are presented in the Experimental section) is amorphous.^27^ A two-hour annealing (450 °C in air) burns the remaining electrolyte and synchronously transforms amorphous TiO_2_M to anatase crystalline.^28^Fig. 1A shows the scanning electron microscopy (SEM) images of the as-formed TiO_2_M, composed of ∼48 μm thick parallel nanochannels (inset of Fig. 1A). Two entrances of nanochannels have different diameters, showing ∼150 ± 20 nm (denoted as the base entrance, Fig. 1A) at one side and ∼40 ± 10 nm at the other (denoted as the tip entrance, Fig. 1B). To achieve an asymmetrical decorated CuMOF for chiral sensing, CuNPs are first coated onto the tip entrance by utilizing the photocatalytic activity of TiO_2_ nanochannels and interfacial growth strategy (details are presented in the Experimental section).^29^Fig. 1C shows that a high-density layer of CuNPs is distributed on the tip entrance and nanochannel walls. These CuNPs are then transferred to homochiral MOFs exhibiting a mesoporous structure (Meso-l-CuMOF) by a typical template-assisted synthesis method (using BDC as an organic ligand, l-Phe as a chiral environment, and CTAB as a template).^30^ As shown in Fig. 1D and F, a layer of compact Meso-l-CuMOF nanocrystals appears on the tip entrance of TiO_2_M with a thickness of ∼1.5 μm (further morphology details are presented in Fig. S1†).

**Fig. 2:**
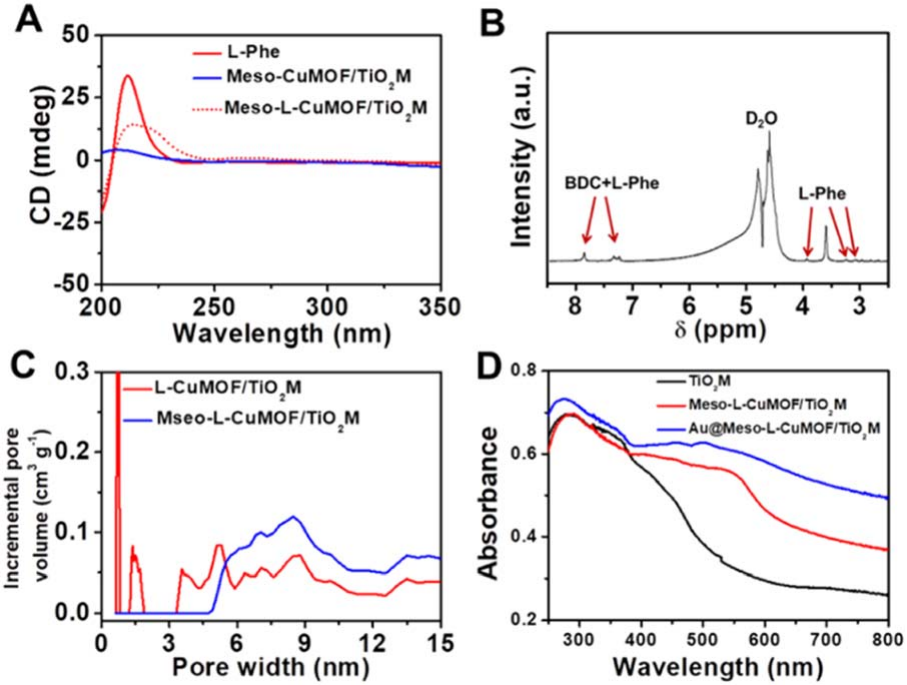
(A) CD spectra of Meso-l-CuMOF/TiO_2_M, l-Phe, and Meso-l-CuMOF/TiO_2_M. (B) Liquid ^1^H NMR spectrum of Meso-l-CuMOF/TiO_2_M digested in D_2_O. (C) Pore size distribution of Meso-l-CuMOF/TiO_2_M before and after CTAB removal. (D) Absorption spectra of TiO_2_M, Meso-l-CuMOF/TiO_2_M, and Au@Meso-l-CuMOF/TiO_2_M.

The sample's circular dichroism (CD) spectrum is recorded and compared with that of the model prepared without l-Phe (named Meso-CuMOF/TiO_2_M) to identify the homochiral character of the resulting MOF layer. Fig. 2A shows an adsorption peak at 210 nm on the Meso-l-CuMOF/TiO_2_M CD spectrum, indicating the homochirality feature. The liquid ^1^H NMR spectrum of Meso-l-CuMOF/TiO_2_M also demonstrates the successful loading of l-Phe into the Meso-CuMOF (Fig. 2B). The mass percentage (9.6 wt%) of l-Phe in the membrane is determined by thermogravimetric analysis (TGA, Fig. S2†). Compared with the sample containing the CTAB templates (named l-CuMOF/TiO_2_M), the content of mesopores in Meso-l-CuMOF/TiO_2_M increases as indicated by the enhanced intensity at a pore size of ∼7.5 nm (Fig. 2C), confirming the presence of a mesoporous structure in MOF layers. Meanwhile, the presence of mesopores is also demonstrated by Brunauer–Emmett–Teller (BET) analysis (Fig. S3†). The obtained BET areas are 406 m^2^ g^−1^ and 268 m^2^ g^−1^ for l-CuMOF/TiO_2_M and Meso-l-CuMOF/TiO_2_M, respectively.^31^ The reduced BET area can be ascribed to the lost micropores. The accommodation space of mesopores provides a favorable feature for further functionalization. To further confirm the successful removal of CTAB, the Raman and energy dispersive X-ray spectroscopy (EDS) analysis of l-CuMOF/TiO_2_M and Meso-l-CuMOF/TiO_2_M were performed. Fig. S4† shows the Raman spectra. The disappearance of CH_3_ (from the (CH_3_)_3_N^+^ group in CTAB) rocking bands at 758 and 1526 cm^−1^ implies the successful removal of CTAB.^32^ Meanwhile, the removal of CTAB is also demonstrated by EDS analysis (Fig. S5†). The reduced N and C elements can be ascribed to the lost CTAB molecules. In this work, the well-known oxidase-mimicking AuNPs are integrated into Meso-l-CuMOF *via* a typical AuCl_4_^−^ adsorption-NaBH_4_ reduction method (Scheme 1).^33^ As indicated by transmission electron microscopy (TEM) images, the nanochannel wall is coated with a MOF layer (Fig. S6†). As shown in Fig. 1F, the high-angle annular dark-field scanning TEM (HAADF-STEM) image shows that AuNPs are well dispersed on Meso-l-CuMOF/TiO_2_M, and the diameter of AuNPs is 5 ± 2 nm (Fig. 1G). The high-resolution TEM (HRTEM) images in Fig. 1H and I exhibit the characteristic lattice spacing for the (200) plane of AuNPs (JCPDS no. 4-784) and the (101) plane of anatase TiO_2_ (JCPDS no. 21-1272). The EDS mapping images reveal the well-dispersed Au, Cu, and N elements on channel walls (Fig. 1I). As the N resource only stems from the –NH_2_ group in l-Phe, this result also verifies the successful fabrication of Au@Meso-l-CuMOF in TiO_2_M. An increased absorption peak at ∼400–700 nm is observed in the UV-vis absorption spectra (Fig. 2D), assigned to the optical absorption of scattered light by the LSPR effect of AuNPs,^34^ indicating an improved light-harvesting ability in the visible-light region. To further demonstrate successful formation of the AuNPs with an according LSPR, we peeled off AuNPs from the Au@Meso-l-CuMOF/TiO_2_M sample by dissolving the Meso-l-CuMOF/TiO_2_M substrate in a HF solution. The resulting solution shows a pink color with an absorption peak at ∼530 nm typical for the SPR of AuNPs (Fig. S7†). In addition, the whole fabrication process is characterized by Fourier transform infrared (FTIR) spectra (Fig. S8†) and X-ray diffraction (XRD) patterns (Fig. S9†). The absorption bands at ∼3065, 1670, 1265, and 1050 cm^−1^ in the FTIR spectra coincide with those of bulk Meso-l-CuMOF powder. The adsorption band at 1265 cm^−1^ corresponds to the C–N stretching of l-Phe. In the XRD patterns, the appearance of Meso-l-CuMOF peaks at 7.5°, and the disappearance of CuNP peaks at 42.5° and 43.5° also implies the transfer of CuNPs to Meso-l-CuMOF. The characteristic diffraction peak at 44.6° is attributed to the Au (200) lattice place, confirming the successful introduction of Au.

**Fig. 3:**
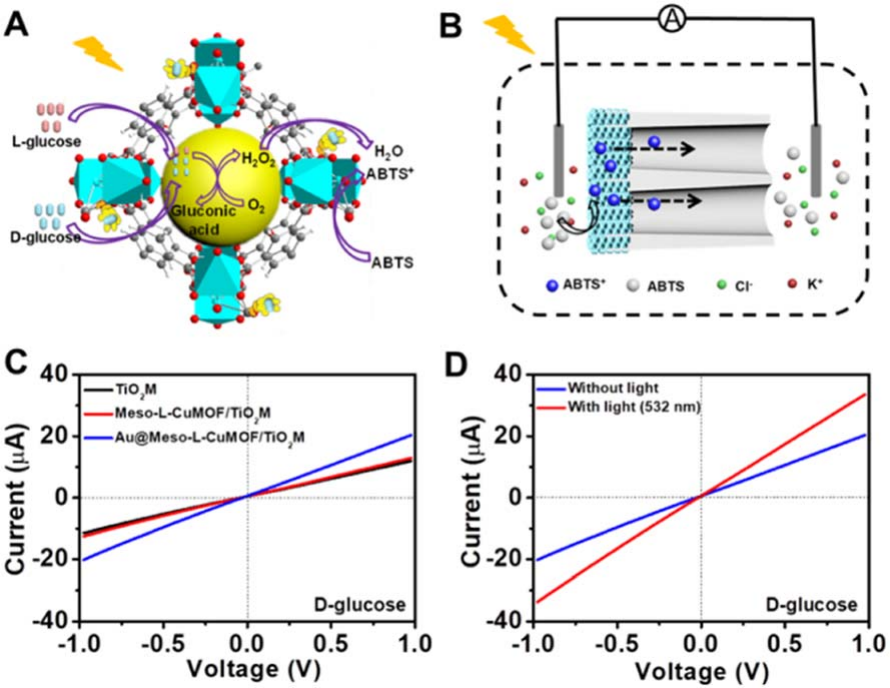
(A) Schematic illustration of enantioselective glucose detection based on cascade reactions. (B) Schematic setup for enantioselective glucose detection based on cascade reactions. (C) *I*–*V* curves of TiO_2_M, Meso-l-CuMOF/TiO_2_M, and Au@Meso-l-CuMOF/TiO_2_M in 10 μM d-glucose. (D) *I*–*V* curves of Au@Meso-l-CuMOF/TiO_2_M for 10 μM d-glucose with or without 532 nm laser irradiation.

A target-discrimination-induced signal-on sensing strategy is designed by integrating the cascade reactions catalyzed by oxidase-mimicking AuNPs and the subsequent Fenton-like CuMOF to achieve the recognition and quantification of glucose enantiomers in the as-proposed membrane. As illustrated in Fig. 3A, the glucose enantiomers selectively recognized on homochiral MOFs are transported into the mesopores and then oxidized to gluconic acid and H_2_O_2_*via* oxidase-mimicking AuNPs.^35,36^ ABTS, one of the widely applied probes for evaluating ·OH radicals, is employed as the signal reporter to provide a noticeable ionic current change. Owing to the Fenton-like performance of Cu(ii),^17^ the CuMOF catalyzes the decomposition of H_2_O_2_ molecules into ·OH radicals, further oxidizing ABTS to ABTS^+^. In addition, the Fenton-like activity of Cu(ii) has been demonstrated to rely on the solution pH.^17^ The decrease in ambient pH caused by gluconic acid generated in the glucose oxidation reaction is thus favorable for activating the Fenton-like activity of the CuMOF. The generated ABTS^+^ cations change the ionic flux, as illustrated in Fig. 3B. The *I*–*V* properties of nanochannels thus offer sensitive signal transduction, indicating the corresponding details for enantiomer recognition and quantification. A traditional colorimetric assay studies the feasibility of the above hypothesis essay based on the characteristic blue color and the typical absorption peak of ABTS^+^ at 400–900 nm (Fig. S10†). By adding H_2_O_2_, the apparent blue color and characteristic absorption peak of ABTS^+^are observed in Meso-l-CuMOF/TiO_2_M (Fig. S10A†), verifying the Fenton-like performance of the CuMOF. In addition, when replacing H_2_O_2_ with d-glucose, the formation of ABTS^+^ is only found in Au@Meso-l-CuMOF/TiO_2_M (Fig. S10B†), verifying the oxidase-mimicking activity of the decorated Au NPs. Noticeably, the absorbance intensity of ABTS^+^ is discriminatory in the presence of glucose enantiomers (Fig. S11†). d-Glucose induces more ABTS^+^ generation than l-glucose at the same concentration, suggesting a higher affinity between Au@Meso-l-CuMOF/TiO_2_M and d-glucose.

Encouraged by the difference in oxidation ability of nanochannels towards glucose enantiomers and their ionic transport feature, the enantiomer recognition and glucose oxidation kinetics of Au@Meso-l-CuMOF/TiO_2_M are investigated by using the ionic transmembrane currents (*I*–*V* curves). The as-prepared membrane is in the middle of the “H-type” cell, as illustrated in Fig. 3B. Two Ag/AgCl electrodes are inserted into two-half cells containing the same electrolyte (1.0 μM KCl containing 1.0 mM ABTS). The ionic transmembrane currents are recorded on TiO_2_M, Meso-l-CuMOF/TiO_2_M, and Au@Meso-l-CuMOF/TiO_2_M (Fig. 3C). The current enhancement is observed in Au@Meso-l-CuMOF/TiO_2_M. The increased ionic flux indicates the formation of ABTS^+^ ions, and the ionic current results coincide with the results of the colorimetric assays (Fig. S10B†).

The Fenton or Fenton-like process decomposes H_2_O_2_ molecules and generates ·OH radicals. In our design, as the yield of ABTS^+^ ions is directly decided by the ionic flux (sensing performance), a larger amount of ·OH radicals is favorable. Recent reports show that the excited LSPR state of a plasmonic metal (mainly Au, Ag, and Pt) effectively facilitates the generation of hot electrons, subsequently transferred from the plasmonic metal to adsorbed reactants, thus elevating the enzyme-mimicking catalytic performance.^37–39^ The glucose oxidation in Au@Meso-l-CuMOF/TiO_2_M nanochannels is investigated under visible-light irradiation (532 nm laser). When exposed to light, an apparent increase in ionic current appears (Fig. 3D, red line). The increased oxidase-mimicking catalytic activity of Au@Meso-l-CuMOF/TiO_2_M under light irradiation is ascribed to the improved light-scattering ability of Au-LSPR (Fig. 3D and S12†). The colorimetric assays are further investigated at 25 and 60 °C to exclude the thermal effect on catalytic activity under laser irradiation (Fig. S13†). Although the ABTS^+^ products generated at 60 °C are slightly larger than that at 25 °C, higher ABTS^+^ yields are observed for both colorimetric assays when systems are exposed to a 532 nm laser. These results confirm that the enhanced ionic current mainly stems from the plasmon-enhanced oxidase-mimicking activity.

**Fig. 4:**
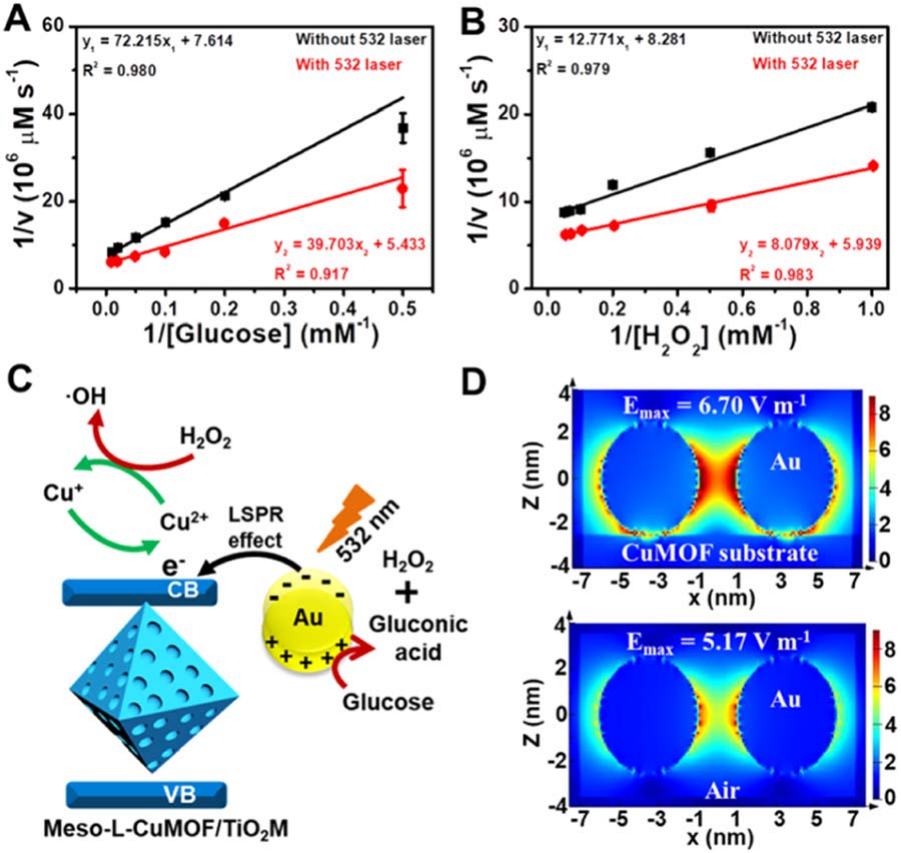
Lineweaver–Burk plot of Au@Meso-l-CuMOF/TiO_2_M for the changes in (A) glucose and (B) H_2_O_2_ in the presence and absence of 532 nm irradiation. (C) Schematic illustration of surface plasmon-enhanced nanoenzyme activity under 532 nm irradiation. (D) The intensity of electric field distribution in the monitor window around the hotspot on Au/CuMOF and Au NPs.

Considering the ionic current variation in the oxidase-mimicking activity of AuNPs and the Fenton-like activity of CuMOFs, the LSPR-enhanced catalytic activity of Au@Meso-l-CuMOF/TiO_2_M is explored from these two aspects. The reaction kinetics of glucose oxidation is further determined in the presence and absence of a 532 nm laser (Fig. S14A and B†) to gain insight into the LSPR effect on the oxidase-mimicking activity of AuNPs. Fig. 4A and S14C† depict the corresponding Lineweaver–Burk plots towards different concentrations of d-glucose and ABTS, respectively. Upon LSPR excitation, the catalytic efficiency (*K*_cat_/*K*_m_) is increased 1.82 and 3.07 times for d-glucose and ABTS, respectively (Table S1†), confirming the LSPR-enhanced oxidase-mimicking activity. The plasmon-enhanced Fenton-like activity of Meso-l-CuMOF is also investigated. The reaction kinetics for H_2_O_2_ decomposition by Fenton-like catalysis is also evaluated (Fig. S15†). The steady-state kinetic parameters of the Fenton-like activity of Meso-l-CuMOF/TiO_2_M for H_2_O_2_ are shown in Fig. 4B, suggesting an effective reaction rate under irradiation. Upon LSPR excitation, the catalytic efficiency increases by 1.74 and 1.55 for ABTS and H_2_O_2_, respectively (Table S2†), demonstrating the LSPR-enhanced Fenton-like activity. These enhancements are attributed to the oscillating energetic hot spots (including “hot holes” and “hot electrons”) generated from LSPR-induced charge separation on a plasmonic metal surface. The hot holes are driven to the AuNP surface by the local electromagnetic fields and then participate in the glucose oxidation,^38–40^ thus leading to the enhanced production of H_2_O_2_, as shown in Fig. 4C.

Due to energy matching, the energetic hot electrons are injected into the conduction band (CB) of CuMOFs, to convert Cu(ii) nodes to Cu(i). The outstanding Fenton-like activity of Cu(i) also accelerates H_2_O_2_ cleavage into ·OH radicals,^41^ thus achieving an improved ABTS yield. In addition, the Fenton-like activity at different temperatures is also carried out to show that the increased surface temperature does not induce enhanced activity under laser irradiation (the sample temperature exhibits a ∼7 °C enhancement after 30 min of 532 nm laser irradiation, Fig. S16†). Comparing the colorimetric assays (Fig. S17†) and *I*–*V* properties (Fig. S18†) at 25 °C and 60 °C, the heating influence on Fenton-like performance of Meso-l-CuMOF/TiO_2_M is still limited, implying that the increased ABTS^+^ yield is mainly attributed to the formation of Cu(i). To further confirm the increased ionic transport performance result after hot injection, the *I*–*V* properties of Au@Meso-l-CuMOF/TiO_2_M at 32 °C (increased by 7 °C from 25 °C) and 67 °C (increased by 7 °C from 60 °C) were tested and compared with the *I*–*V* curves measured after 532 nm-laser irradiation. As shown in Fig. S19A,† a slight current enhancement is observed when the temperature increased from 25 °C to 32 °C. When exposed to light (523 nm laser), a more apparent enhancement in ionic current is observed (Fig. S19A†). As shown in Fig. S19B,† the ionic current enhancement is ignored as the temperature increased from 60 °C to 67 °C, which can be attributed to the stable GOD-like activity of AuNPs at higher temperature.^42^ In contrast, an apparent increase in ionic current appears when exposed to light (Fig. S19B†). The above results demonstrate that the increased ionic transmembrane current is related to the hot injection and increased temperature at a lower temperature, and is mainly caused by hot injection at a higher temperature. These results indicate that the formed energetic charge carriers improve oxidase-mimicking and Fenton-like activity upon LSPR excitation.

Besides the dielectric constant of the plasmonic metal, the LSPR intensity of plasmonic nanoparticles is related to many factors, such as the particles' size, shape, and density, as well as the dielectric environment.^43,44^ To gain additional evidence of energetic hot spot generation by LSPR of AuNPs in the Meso-l-CuMOF, the finite difference time domain (FDTD) method is employed to investigate the field strength distribution in the Au@Meso-l-CuMOF structure, based on the TEM images in Fig. S6† (the permittivity of gold used in the simulation is obtained from Palik's data^45^). Besides the plasmonic enhancement in the nanogap between two Au nanoparticles, LSPR intensity shows an apparent increase at the interface between AuNPs and the Meso-l-CuMOF substrate (Fig. 4D). Moreover, according to a recent report by Beckham^46^ and Tiefenbacher,^47^ the nanoconfinement effect of supramolecular hosts also facilitates the dual activation of both a nucleophilic (*i.e.*, glucose oxidation) and an electrophilic (*i.e.*, H_2_O_2_ cleavage) reaction in isolated binding pockets (*i.e.*, the well-aligned mesoporous MOFs). These results indicate that the dielectric CuMOF substrate and the mesoporous frame structure favor the energetic charge carrier generation process.

**Fig. 5:**
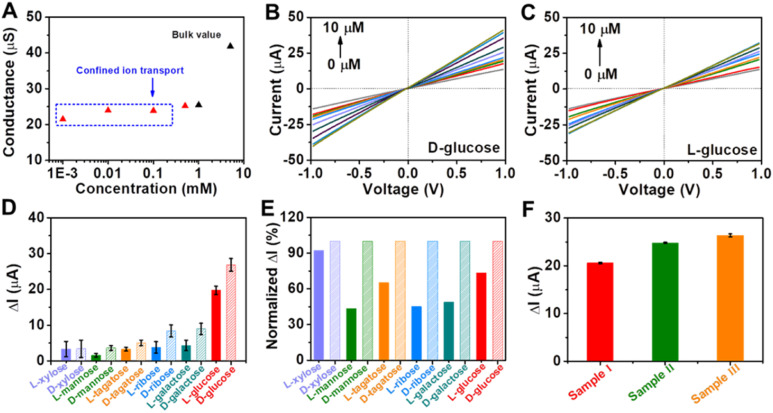
(A) Ionic conductance of the membrane as a function of KCl concentrations. *I*–*V* curves for sensing different concentrations of (B) d-glucose and (C) l-glucose. (D) Ionic and (E) normalized ionic current changes at +1.0 V for monosaccharide enantiomers (10 μM). (F) Detection of d-glucose in serum samples. The electrolyte contains 1.0 mM ABTS and 1.0 μM KCl (pH 5.0).

Encouraged by the remarkable catalytic activity of Au@Meso-l-CuMOF/TiO_2_M for glucose oxidation and the homochiral character of nanochannels, the *I*–*V* properties of the resulting membrane are applied to discriminate between glucose enantiomers. The transmembrane ionic conductances are investigated at different KCl concentrations to evaluate the ionic transport performance of Au@Meso-l-CuMOF/TiO_2_M (Fig. S20†). The ionic conductance is mainly attributed to the bulk when the concentrations exceed 1.0 mM, as presented in Fig. 5A. When the concentrations are lower than 1.0 mM, the ionic conductance deviates from the bulk value, attributed to the electric double layer (EDL)-governed ionic transport in mesopores at low concentrations.^48,49^ To enable the ion current which mainly stems from ABTS^+^ ions, a low-concentrated KCl (1.0 μM) solution is chosen for l/d-glucose recognition and detection. For high detection sensitivity, the operating parameters, such as the concentration of ABTS, reaction temperature, and pH, are optimized through *I*–*V* curves of Au@Meso-l-CuMOF/TiO_2_M under light irradiation. The ABTS concentration is optimized as 1.0 mM based on the change in the *I*–*V* curves (Fig. S21†). The reaction temperature is studied from 25–80 °C, and the highest catalytic activity is obtained at 60 °C (Fig. S22†). The pH effect on cascade reactions is also evaluated based on the *I*–*V* curves (Fig. S23†). A burst ABTS production is found at pH 5.0. Accordingly, *I*–*V* measurements in the following study are performed in KCl solution (1.0 μM, pH 5) containing 1.0 mM ABTS at 60 °C. However, considering high temperatures largely influence the hydrogen bonds formed in the enantiomeric identification process,^50^ recognition is achieved at 4 °C for 4 h to gain a saturated adsorption state (Fig. S24†). The nanochannels exhibit negligible catalytic activity at 4 °C, and recognition at 4 °C reaches a saturated state after 4 h. Therefore, before triggering the glucose oxidation reaction at 60 °C, enantiomer recognition is performed at 4 °C for 4 h. The discrimination abilities of Au@Meso-l-CuMOF/TiO_2_M and Au@Meso-CuMOF/TiO_2_M for 10 μM of glucose enantiomers are evaluated through *I*–*V* curves under optimal conditions. Fig. S25A† shows the *I*–*V* curves of Au@Meso-l-CuMOF/TiO_2_M after cascade reactions catalyzed by enzymatic-mimicking for 10 μM l-glucose and 10 μM d-glucose. Apparently, the ionic current elevation induced by 10 μM d-glucose is larger than that of 10 μM l-glucose. For comparison, the *I*–*V* curves of Au@Meso-CuMOF/TiO_2_M were also recorded (Fig. S25B†), which showed a similar ionic current elevation for the recognition of l/d-glucose. The above results demonstrate that the enantioselectivity stems from the chiral environment induced by l-CuMOF. To further confirm this conclusion, we also employed colorimetric assay to investigate the effect of Au NPs on selectivity. The oxidation ability of Au@Meso-CuMOF/TiO_2_M to l/d-glucose can be indicated by the color change of ABTS solution (the generated H_2_O_2_ would oxidize ABTS to form blue colored ABTS^+^). As shown in Fig. S25C and D,† the results are consistent with the *I*–*V* curves. The l-Phe molecules act as enantioselective identifiers to interact with d-glucose than with l-glucose *via* hydrogen-bonding interactions.^51^ The discrimination abilities of Au@Meso-l-CuMOF/TiO_2_M for different concentration of glucose enantiomers are evaluated through *I*–*V* curves under optimal conditions (Fig. S26 and S27†). Fig. 5B and C plot the *I*–*V* curves of different concentrations of d-glucose and l-glucose at a 30 min-reaction period at 60 °C, respectively. The ionic currents gradually increase with glucose concentration from 0.1 to 10 μM. The ionic current elevation induced by d-glucose is larger than that of l-glucose from the ionic current change (Δ*I*) at different glucose concentrations (Fig. S28†). The calibration curves show that the resulting Au@Meso-l-CuMOF/TiO_2_M has an excellent linear response to d-glucose sensing over two ranges: from 0.1 μM to 1 μM and from 1.0 μM to 10 μM. The limit of detection (LOD) is 0.089 μM, based on a 3 SD/L method. Compared with other reported recognition methods (Table S3†), this strategy exhibits a better LOD value, comparable with the recently reported colorimetric assay,^52^ CD spectra,^53^ and electrochemical methods.^54^

Application versatility and enantioselectivity are essential for evaluating a chiral recognition platform. The chiral sensing possibility of Au@Meso-l-CuMOF/TiO_2_M for monosaccharide enantiomers is estimated (Fig. S29†). Fig. 5D plots the ionic current changes of the membrane for l/d-mannose, l/d-xylose, l/d-tagatose, l/d-ribose and l/d-galactose discrimination. The glucose enantiomers show a more significant relative ionic current variation, attributed to the substrate selectivity of oxidase-mimicking AuNPs, also discovered in a previous report.^55^Fig. 5E further presents the normalized ionic current changes. The chiral sensing device exhibits an excellent stereochemically controlled reaction process; d-monosaccharides show a more significant affinity for chiral MOFs.

Considering the promising applications in biological samples, the main interfering species co-existing with d-glucose in human blood serum are investigated (Fig. S30†). The ionic current changes induced by these interferences are ignored under the same experimental conditions as those of d-glucose sensing. Encouraged by the above results, the detection of d-glucose is further investigated in serum samples (Fig. 5F and S31†). The glucose concentrations determined from Δ*I* values (Table S4†) show satisfactory recovery with a low relative standard deviation (RSD), consistent with the hospital results obtained by a classic glucose oxidase–peroxidase method.

The finite element method (FEM) simulation is further applied to gain insight into the reactant and ionic product distribution in nanochannels to understand the mechanism of the high sensitivity of the proposed sensing system. A theoretical model (Fig. S32 and Table S5†) based on FEM combined with Poisson and Nernst–Planck (PNP) equations^56,57^ (details are provided in the ESI†) is employed to simulate the glucose sensing process in Au@Meso-l-CuMOF. Upon LSPR excitation, AuNPs catalyze glucose oxidation to H_2_O_2_ and gluconic acid. The reaction kinetics, eqn (1) and (2), can be formulated according to the Michaelis–Menten equation.1
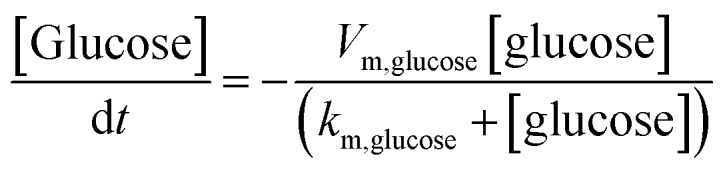
2
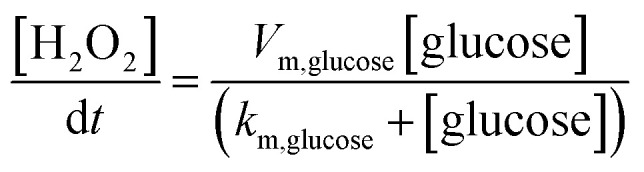


The reaction intermediate H_2_O_2_ is subsequently cleaved to ·OH radicals by CuMOF with the aid of hot electrons. The kinetics are expressed as follows:3
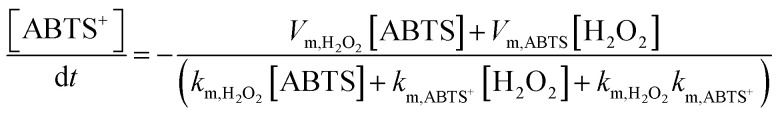


**Fig. 6:**
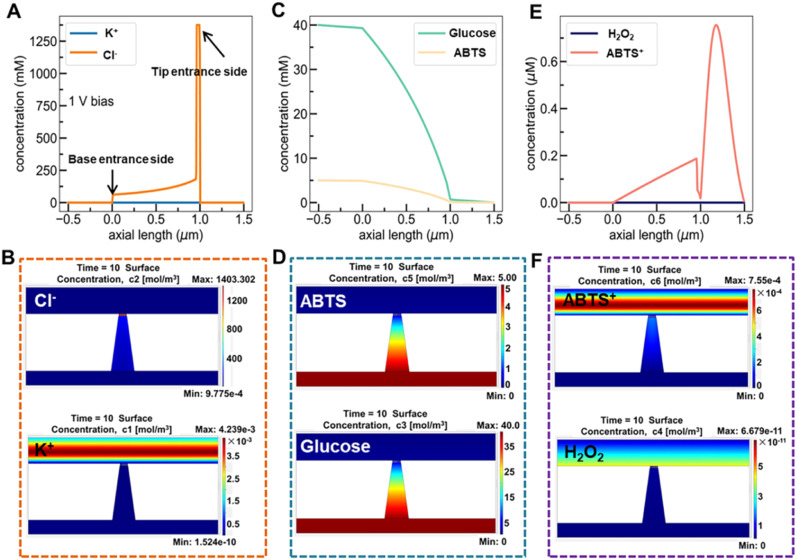
(A) Simulation concentration profiles of KCl along a nanochannel without an enzyme catalytic reaction. (B) Simulated ionic concentration images of Cl^−^ and K^+^ ions in a nanochannel without an enzyme catalytic reaction. (C) Simulation concentration profiles of glucose and ABTS along the nanochannel. (D) Simulated ionic concentration images of glucose and ABTS in a nanochannel. (E) Simulation concentration profiles of H_2_O_2_ and ABTS^+^ along the nanochannel. (F) Simulated ionic concentration images of H_2_O_2_ and ABTS^+^ in a nanochannel. The transmembrane voltage is set as +1.0 V.

Fig. 6A and B show the simulated distribution of K^+^ and Cl^−^ concentration profiles and the corresponding ionic flux images (at +1.0 V) on the Au@Meso-l-CuMOF/TiO_2_M surface in the absence of glucose. Since the Au@Meso-l-CuMOF/TiO_2_M zone carries positive surface charges (Fig. S33†), the strong electrostatic interactions attract more Cl^−^ ions into the nanochannels, thus accumulating Cl^−^ ions in Au@Meso-l-CuMOF/TiO_2_M. The concentration profiles (Fig. 6C) and corresponding ionic flux images (Fig. 6D) of glucose and ABTS along the nanochannel are also simulated before the reaction. The electroneutral feature of both substrates and the electrical field does not influence their diffusion behaviors. The concentration profiles and corresponding ionic flux images of H_2_O_2_ and ABTS^+^ along the nanochannel after glucose oxidation are also simulated (Fig. 6E and F). Due to the low affinity between glucose and CuMOF pockets (high *K*_m_ constant, compared to other *K*_m_), the generated H_2_O_2_ molecule oxidizes ABTS to ABTS^+^ using a CuMOF catalyst. Since ABTS^+^ ions carry a positive charge, they are depleted from mesoporous MOF pockets under solid electrostatic repulsion, increasing ABTS^+^ ion diffusion current. The ion/molecule concentration distribution on the Au@Meso-l-CuMOF/TiO_2_M surface is also investigated at −1.0 V (Fig. S34†), showing similar results as the distribution at +1.0 V.

## Conclusions

In summary, a highly enantioselective and sensitive platform for monosaccharide sensing is designed by the asymmetric assembly of Au@Meso-l-CuMOFs on TiO_2_M. Benefitting from the dual catalytic activity and homochiral environment, the ionic transmembrane currents of nanochannels provide excellent discrimination for monosaccharide enantiomers. Owing to the confinement effect, the primarily generated energetic “hot holes” and “hot electrons” under LSPR excitation in the nanoscaled MOF pocket significantly accelerated the sensing performance. The photocatalysis-triggered asymmetrical decoration approach can be easily extended to preparing many MOF membranes on porous TiO_2_ substrates. This work provides a strategy to acquire and improve the signal for enantioselective recognition.

## Experimental

### Preparation of CuNPs/TiO_2_M

Cu/TiO_2_M was prepared in a self-made H-type cell. The membrane was placed between two cells of a homemade electrolyte cell, and a quartz window was set on one side of the cell (top side of TiO_2_M) to allow UV light to pass through and reach the TiO_2_M surface. One half of the cell (bottom side of the TiO_2_M membrane) was filled with 5.0 mM Cu(CH_3_COO)_2_·H_2_O. Another half of the cell (top side of TiO_2_M) was filled with pure water. At +1.0 V for 120 min, the CuNPs migrated and were then deposited on the top side of the nanochannel, which was exposed to UV light (3 W LED, 365 nm).

### Preparation of Au@Meso-l-CuMOF/TiO_2_M

First, 11.6 mg BDC was dissolved in a 10 mL mixture of DMF and CH_3_OH (V_DMF_:V_CH_3_OH_ = 1 : 1). Known amounts of surfactant CTAB (20 mg) and the chelating agent l-Phe (4.3 mg) were added, and the mixture was sonicated for 30 min to form a homogeneous solution. The as-formed CuNPs/TiO_2_M (diameter 10 mm) was added to the solution. The sealed vessel was held at 80 °C for 24 h for MOF growth in TiO_2_M. The resulting chiral CuMOF/TiO_2_M sample was carefully washed with DMF and CH_3_OH to remove the unreacted ligands and then dried at 80 °C in a vacuum oven to remove the remaining solvent. The as-synthesized sample was extracted twice with an ethanol solution to remove the template (CTAB) from the framework (*t* = 4 h at 60 °C). The Meso-l-CuMOF/TiO_2_M was incubated in a solution of HAuCl_4_ (0.5 mM) at 70 °C for 2 h. After cleaning with DI water, the sample was dipped in a 0.1 mM NaBH_4_ solution for 15 min to produce Au NPs.

### Electrochemical measurements

*I*–*V* curves of channels were measured using a homemade electrochemical cell. The effective exposed area of samples was 0.8 mm^2^, clamped between two PDMS films and placed between two Teflon cells. Two Ag/AgCl electrodes were used to apply the transmembrane potential and measure the ionic current. The electrochemical detection of molecules was performed in an aqueous solution (60 °C) containing different concentrations of l/d-glucose, 1.0 mM ABTS, and 1.0 μM KCl (pH 5). The ionic current transport through hybrid channels was measured in the voltage range of −1.0 to +1.0 V at a scan rate of 50 mV s^−1^.

### Numerical simulations for ionic transport in a Au@Meso-l-CuMOF/TiO_2_M nanochannel array

The nanofluidic sensor mechanism for glucose was proposed by using a theoretical model and was evaluated by the finite element method (FEM), combined with Poisson and Nernst–Planck (PNP) equations. The equations are shown below:^56,57^4
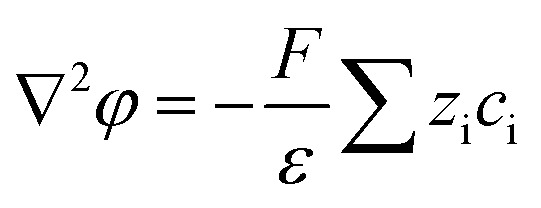
5
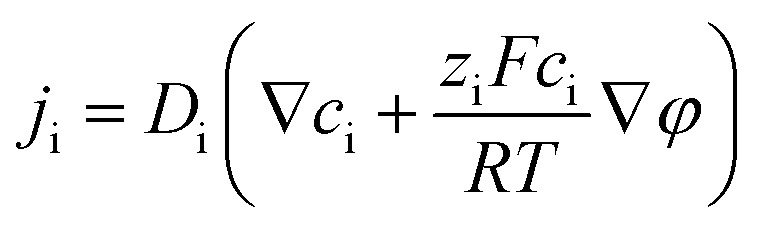

6 ∇*j*_i_ = 0

where *j*_i_, *D*_i_, *C*_i_, *φ*, *R*, *F*, *T*, and *ε* are the ionic flux, diffusion coefficient, ion concentration, electrical potential, universal gas, Faraday constant, absolute temperature, and dielectric constant of electrolyte solution, respectively. Eqn (4), called the Poisson equation, characterized the electric potential and ionic concentration. Eqn (5) is the Nernst–Planck equation, indicating the charged nanochannel transport behavior. Besides, when the system reaches a steady state, the flux should satisfy eqn (6). The coupled eqn (4)–(6) was used to calculate the ion concentration distribution and solved by assuming appropriate boundary conditions.7
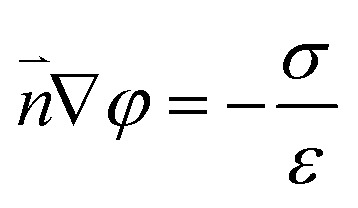
8
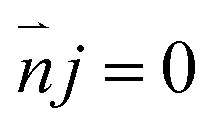


The boundary condition for the potential *φ* on the channel wall was eqn (7), where the ionic flux exhibited zero normal components at boundaries eqn (8).

## Data availability

The data supporting the findings of this study are available within the article and in the ESI.†

## Author contributions

Y.-Y. Song conceived the concept and directed the project. J. L. Guo, X. J. Xu, and J. J. Zhao performed the experiments. Z.-Q. Wu carried out the theoretical study. Z. D. Gao collected and analyzed the data. J. L. Guo prepared the first draft of this manuscript, and all the authors modified the manuscript.

## Conflicts of interest

There are no conflicts to declare.

## Supplementary Material

SC-014-D2SC05784K-s001
