# Supplementary material for: Rational design of mesoporous chiral MOFs as reactive pockets in nanochannels for enzyme-free identification of monosaccharide enantiomers
Source: Chem Sci. 2023 Jan 17;14(7):1742–51. doi: 10.1039/d2sc05784k (PMC9930935; doi:10.1039/d2sc05784k)
Supplement: SC-014-D2SC05784K-s001 [file SC-014-D2SC05784K-s001.pdf]

Electronic Supplementary Material (ESI) for Chemical Science.
This journal is © The Royal Society of Chemistry 2023

Supplementary Information

Rational Design of Mesoporous Chiral MOFs as Reactive Pockets in Nanochannels for Enzyme-Free Identification of Monosaccharide Enantiomers

Junli Guo,[a] Xuao Liu,[a] Junjian Zhao,[a] Huijie Xu,[a] Zhida Gao,[a] Zeng-Qiang Wu,*[b] and Yan-Yan Song*[a]

[a]College of Sciences, Northeastern University, Shenyang 110819, China

[b]School of Public Health, Nantong University, Nantong, 226019, China

Corresponding Authors

E-mail: yysong@mail.neu.edu.cn

E-mail: zqwu@ntu.edu.cn

EXPERIMENTAL SECTION

Chemicals and reagents. Ti sheets (0.1 mm thickness, 99.6% purity) were purchased from Baosheng Hardware (Bao ji). 1,4-dicarboxybenzene (BDC), Cupric acetate monohydrate ($Cu(CH_3COO)_2 \cdot H_2O$), Hexadecyltrimethylammonium bromide (CTAB), L-Phenylalanine (L-Phe) were purchased from Aladdin. Ammonium fluoride (NH_4F), ethylene glycol, N, N-dimethylformamide (DMF, 99.8%), methanol (CH_3OH), and other chemicals were purchased from Sinopharm Chemical Reagent and used as received without further purification. All aqueous solutions were prepared using Millipore Milli-Q water with a resistivity of 18.0 MΩ·cm.

Apparatus. Morphology was characterized using a field-emission scanning electron microscope (Hitachi SU8000, Japan). A JEOL 2000FX high-resolution transmission electron microscope (HRTEM) (Japan) was used to record TEM and HRTEM images. Crystal structures were identified by XRD acquired using an X'Pert XRD spectrometer (Philips, USA) using a CuKα X-ray source. Zeta potential was measured using a Zetasizer Nano ZS90 analyzer (Malvern, USA). UV-vis absorption spectra were recorded using a Perkin–Elmer spectrometer (Lambda 750S, USA). FTIR spectroscopy was performed using a Nicolet 6700 instrument (Thermo Fisher, USA). Surface-enhanced Raman scattering (SERS) measurements were conducted using a Raman microscopy spectrometer (LabRAMHR, HORIBA Scientific, France). A CHI660D electrochemical workstation (CH Instrument, USA) was used for all electrochemical tests equipped with two Ag/AgCl electrodes as the anode and cathode. Photocatalytic reduction of Cu^{2+} to CuNPs reactions were performed under the irradiation of a ultraviolet LED (3.0 W, wavelength: 365 nm). The enhanced enzyme-like activities were performed under the irradiation of 532 nm laser.

Preparation of TiO_2M.

TiO_2 nanochannel membranes were grown on Ti foils (15 mm × 15 mm × 0.1 mm) by electrochemical anodization. Briefly, Ti foils were first sequentially rinsed with acetone, ethanol, and deionized water and then dried in air. Anodization was carried out in ethylene glycol/lactic acid/water electrolyte containing 0.1 M NH_4F at 120 V and 150 V for 30 min and 2 min, respectively. Samples were dipped in H_2O_2 (30%) until titanium substrates were separated from the membrane to obtain an open-ended TiO_2 nanochannel membrane. The

prepared samples were annealed at 450 °C for 2 h in the air at a heating rate of 3 °C/min.

Preparation of bulk Meso-L-CuMOF.

In a typical synthesis, 0.232 g (1.0 mmol) copper nitrate hemipentahydrate, and 0.116 g (0.56 mmol) BDC were dissolved in a 10 mL mixture of DMF and CH_3OH (V_{DMF}: V_{CH3OH} = 1:1). Then CTAB (0.2 g) and chelating agent L-Phe (0.043 g) were added, and the mixture was sonicated for 30 min to form a homogeneous solution. The solution was heated to 80 °C for 24 h under static conditions. The resulting solid was washed with DMF and CH_3OH to remove unreacted starting materials. Finally, the as-synthesized sample was extracted twice with an ethanol solution to remove the template (CTAB) from the framework (t = 4 h at 60 °C).

Preparation of serum samples.

For determination of glucose in real samples, a commercial serum sample was directly used without pretreatment (Beijing Solarbio Science & Technology Co., Ltd.). A standard addition method was used to evaluate the reliability of the method. The D-glucose concentrations were obtained using the traditional glucose oxidase–peroxidase method. The concentrations of D-glucose in unspiked and spiked serum samples were determined by diluting it to a linear range.

Figures

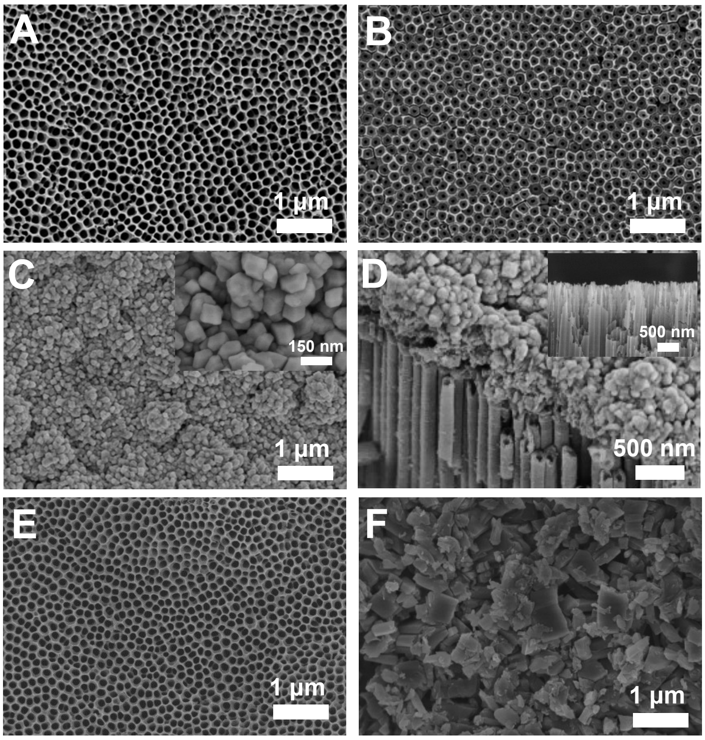

Figure S1. (A) Top-view and (B) bottom-view SEM images of TiO_2M. (C) Bottom-view and (D) side view SEM images of CuNPs/TiO_2M. (E) Top-view SEM images of Meso-L-CuMOF/TiO_2M. (F) Bottom-view SEM images of Meso-L-CuMOF/TiO_2M.

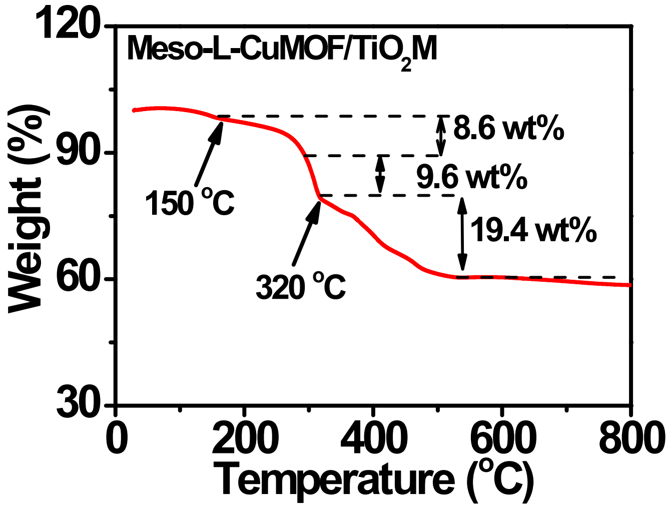

Figure S2. TGA analysis of Meso-L-CuMOF/TiO_2M.

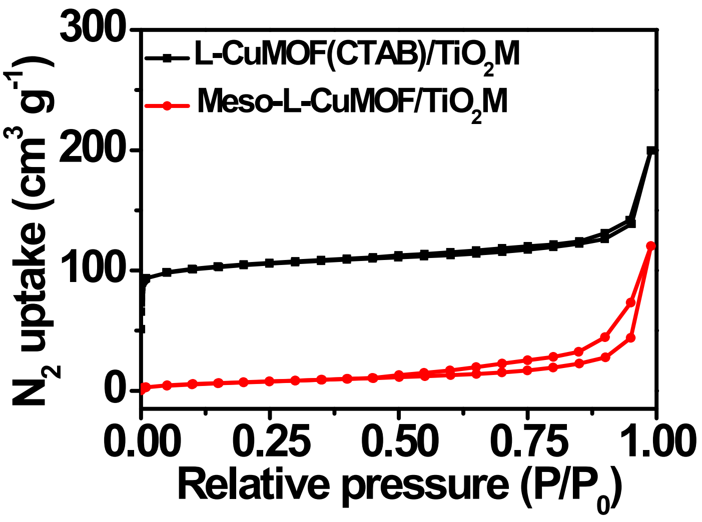

Figure S3. N_2 sorption isotherms of L-CuMOF/TiO_2M before and after CTAB removal.

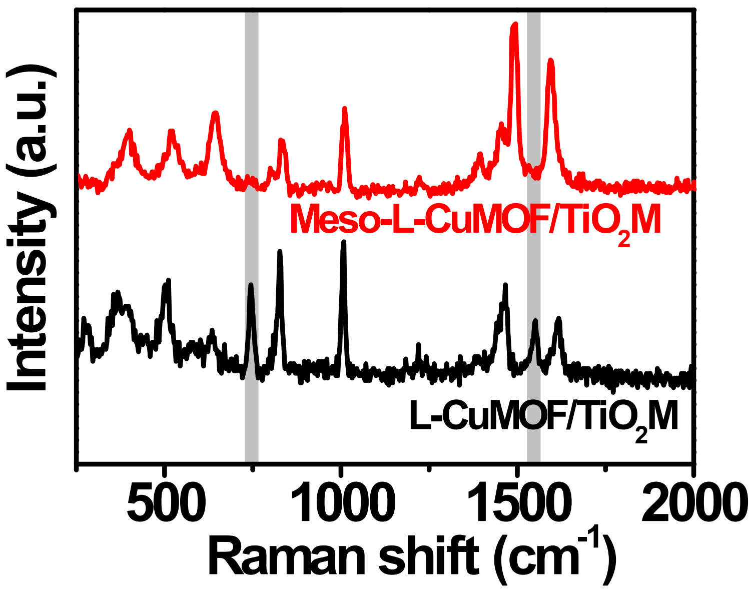

Figure S4. Raman spectra of L-CuMOF/TiO_2M and Meso-L-CuMOF/TiO_2M.

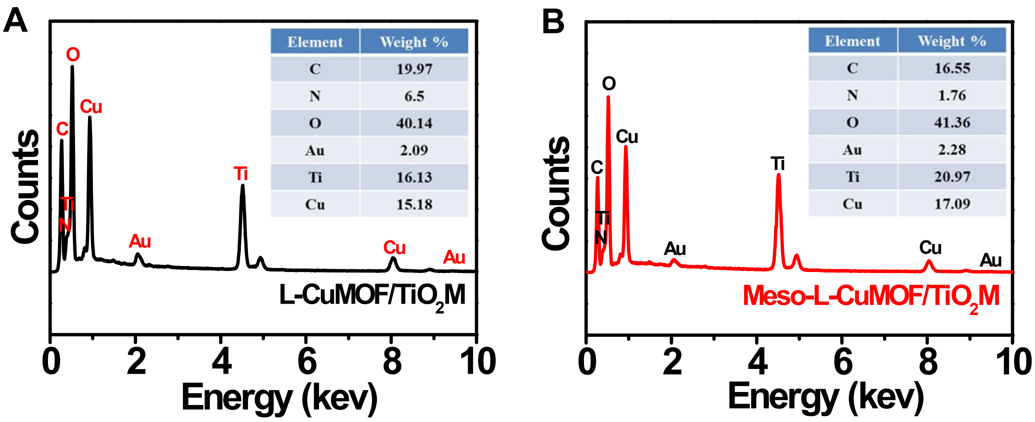

Figure S5. EDS results of (A) L-CuMOF/TiO_2M and (B) Meso-L-CuMOF/TiO_2M.

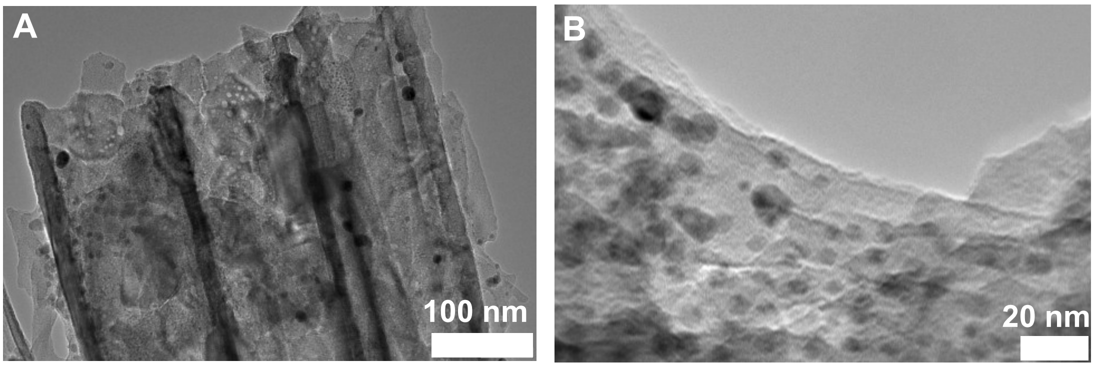

Figure S6. TEM images of Au@Meso-L-CuMOF/TiO_2M.

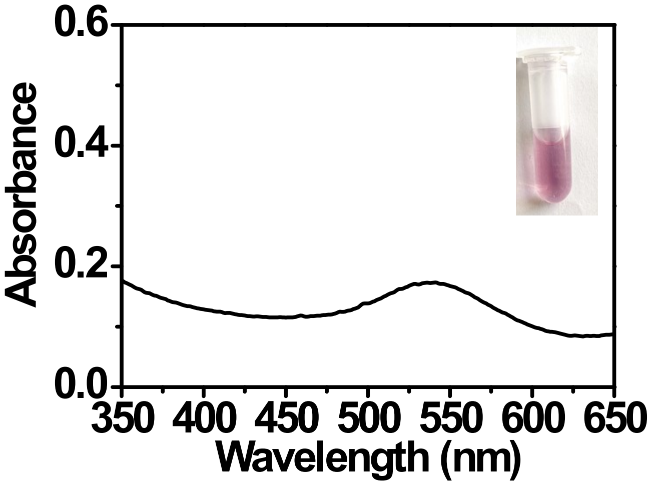

Figure S7. UV-Vis absorption spectrum of AuNPs peeled from Au@Meso-L-CuMOF/TiO_2M. Inset image: the corresponding digital photographs of the AuNPs solution.

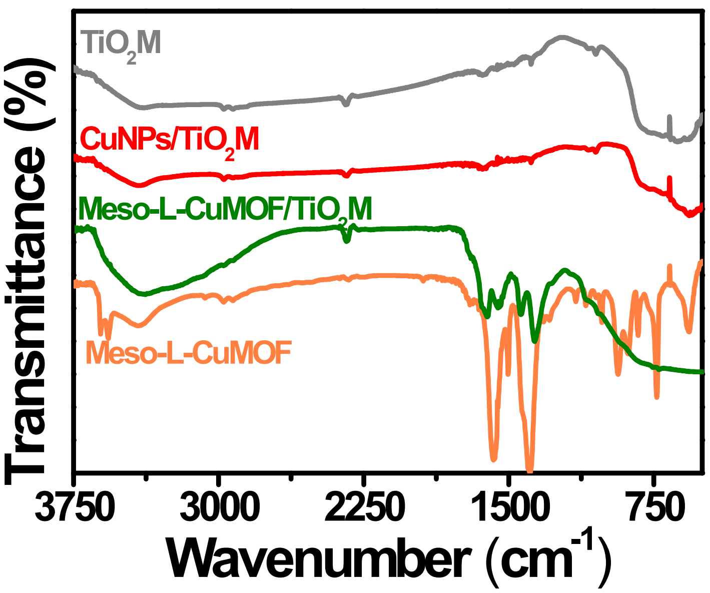

Figure S8. FTIR spectrum of TiO_2M, CuNPs/TiO_2M, Meso-L-CuMOF/TiO_2M, and Meso-L-CuMOF.

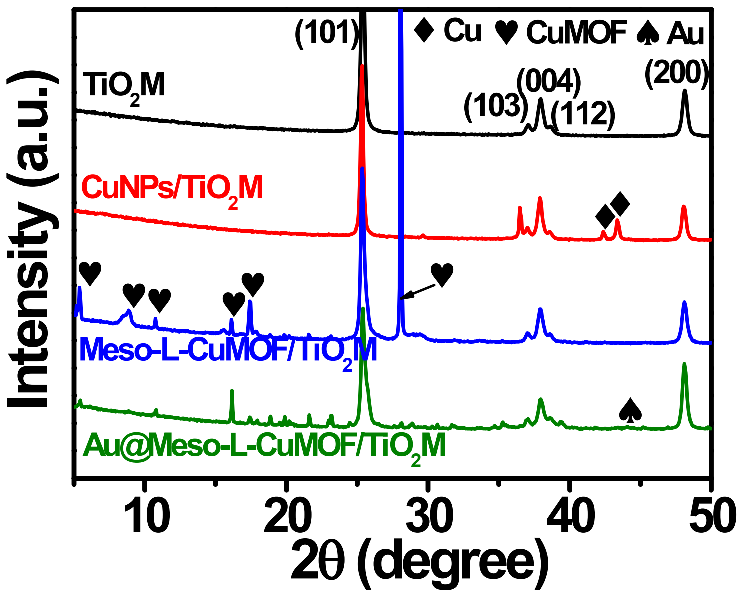

Figure S9. XRD patterns of TiO_2M, CuNPs/TiO_2M, Meso-L-CuMOF/TiO_2M, and Au@Meso-L-CuMOF/TiO_2M.

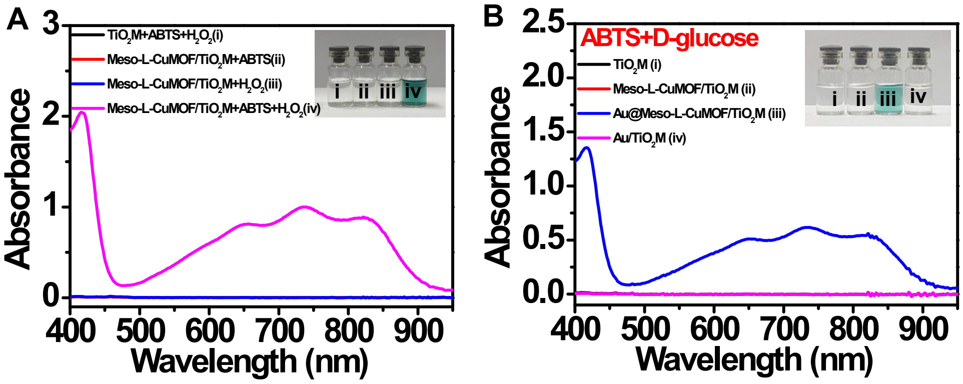

Figure S10. Colorimetric assay of POD-like reaction of Meso-L-CuMOF. (A) UV−vis spectra of different samples: TiO_2M + ABTS + H_2O_2, Meso-L-CuMOF/TiO_2M + ABTS, Meso-L-CuMOF/TiO_2M + H_2O_2, and Meso-L-CuMOF/TiO_2M + ABTS + H_2O_2. Colorimetric assay of glucose-like oxidase activity of Au@Meso-L-CuMOF/TiO_2M. (B) UV−vis spectra of different samples in 1.0 mM D-glucose and 1.0 mM ABTS. Inset image: the corresponding photographs of solutions corresponding to each curve.

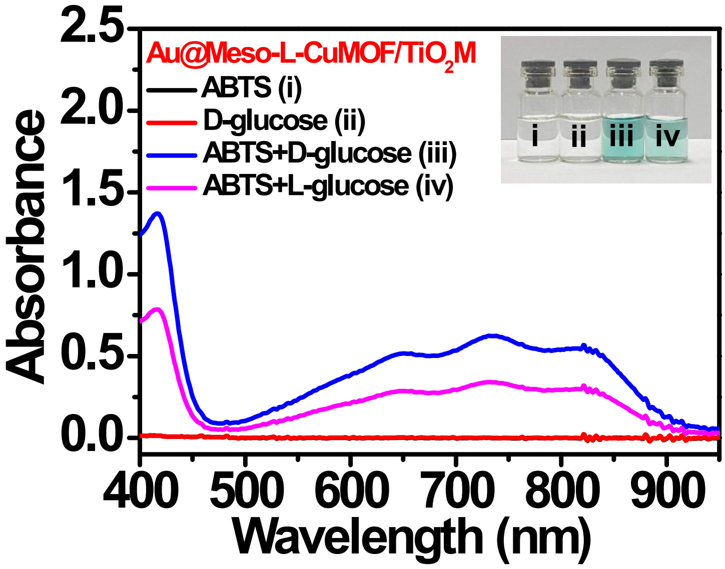

Figure S11. UV−vis spectra of Au@Meso-L-CuMOF/TiO_2M in different solutions. Inset image: the photographs of solutions corresponding to each curve.

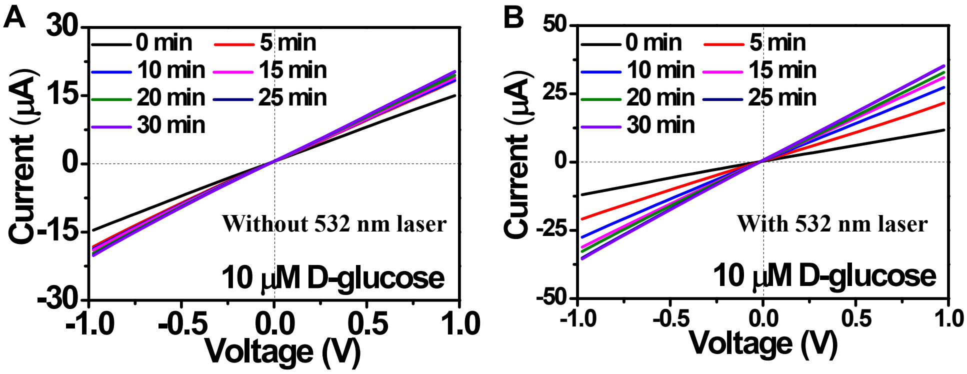

Figure S12. *I-V* properties of Au@Meso-L-CuMOF/TiO_2M in the presence of 10 μM D-glucose without (A) and with (B) 532 nm laser.

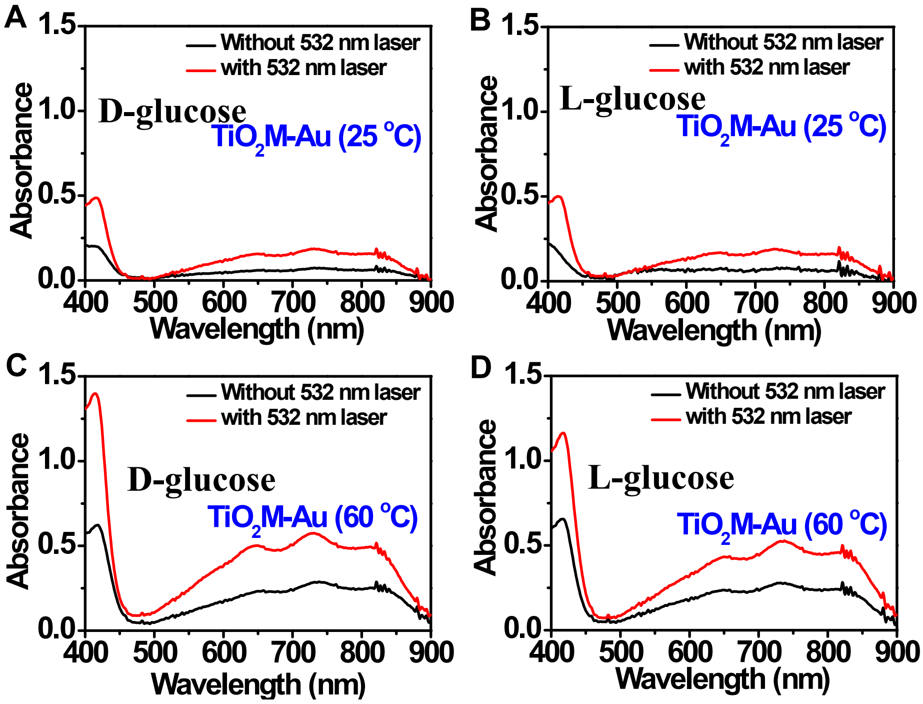

Figure S13. Colorimetric assay of glucose-like oxidase activity of AuNPs for D-glucose (A) and L-glucose (B) at 25 ^{o}C and D-glucose (C) and L-glucose (D) at 60 ^{o}C with and without 532 nm laser irradiation.

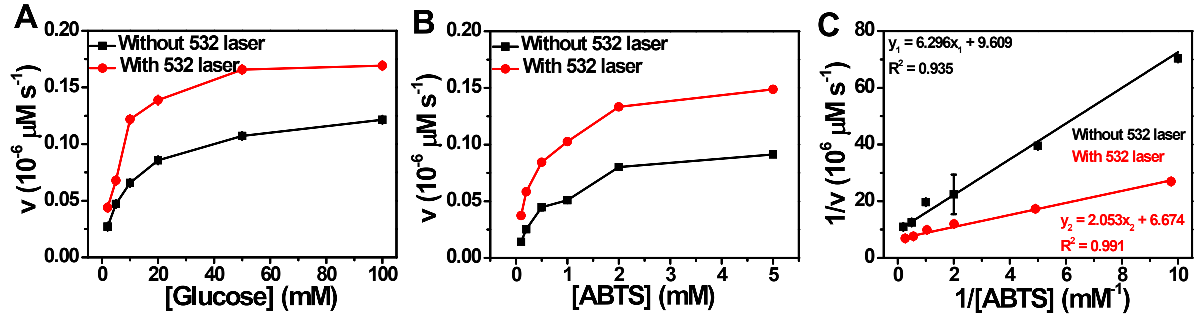

Figure S14. Steady-state kinetic assay for glucose-like oxidase activity of Au@Meso-L-CuMOF/TiO_2M for (A) glucose and (B) ABTS. (C) Lineweaver-Burk plot of Au@Meso-L-CuMOF/TiO_2M by the variation in ABTS.

Table S1. Kinetic parameters for glucose oxidase-like activity of Au@Meso-L-CuMOF/TiO_2M with glucose and ABTS as substrate.

	K_m (mM)	V_{max} (μM/s)	K_{cat} (s^{-1})	K_{cat}/K_m (s^{-1} mM^{-1})
Without irradiation (glucose)	9.485	0.131	6.001	0.633
With irradiation (glucose)	7.308	0.184	8.429	1.153
Without irradiation (ABTS)	0.655	0.104	4.764	7.273
With irradiation (ABTS)	0.308	0.150	6.871	22.308

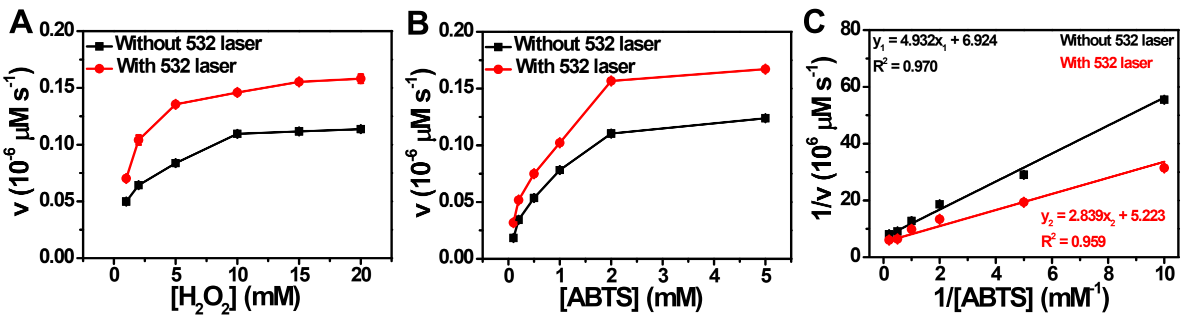

Figure S15. Steady-state kinetic assay for POD-like activity of Au@Meso-L-CuMOF/TiO_2M for (A) H_2O_2 and (B) ABTS. (C) Lineweaver-Burk plot of Au@Meso-L-CuMOF/TiO_2M for the variation in ABTS.

Table S2. Kinetic parameters for the POD-like activity of Au@Meso-L-CuMOF/TiO_2M with H_2O_2 and ABTS as substrate.

	K_m (mM)	V_{max} (μM/s)	K_{cat} (s^{-1})	K_{cat}/K_m (s^{-1} mM^{-1})
Without irradiation (H_2O_2)	1.542	0.121	5.543	3.595
With irradiation (H_2O_2)	1.360	0.168	7.696	5.569
Without irradiation (ABTS)	0.712	0.144	6.596	9.264
With irradiation (ABTS)	0.543	0.191	8.749	16.112

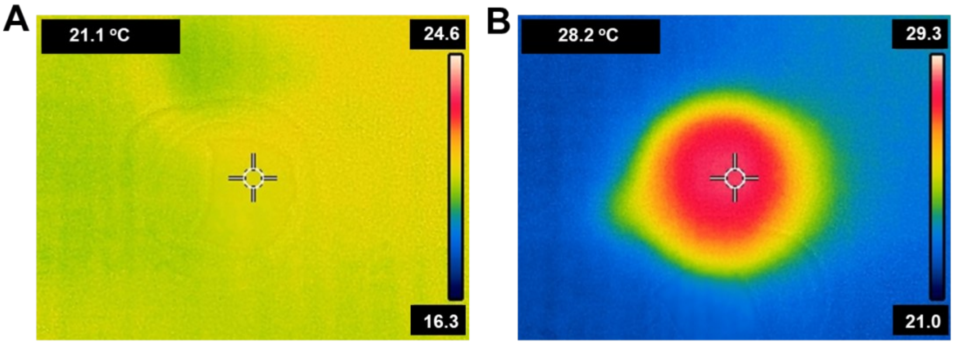

Figure S16. Infrared thermal imaging photographs of Au@Meso-L-CuMOF/TiO_2M under 532-nm laser irradiation for (A) 0 min and (B) 30 min.

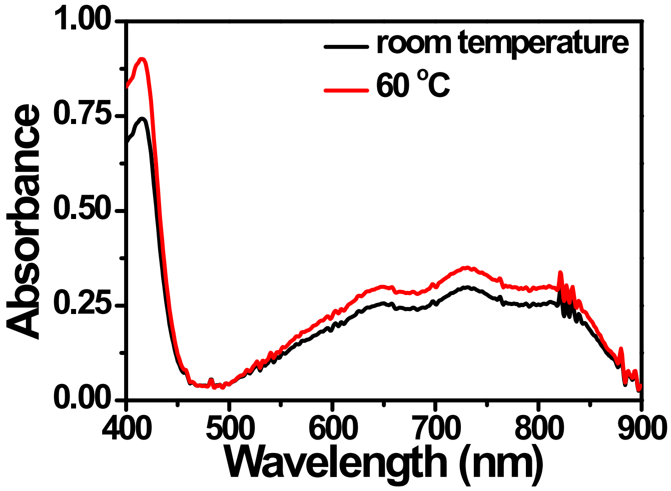

Figure S17. Effect of temperature on Fenton-like activity of Meso-L-CuMOF, determined through colorimetric assay.

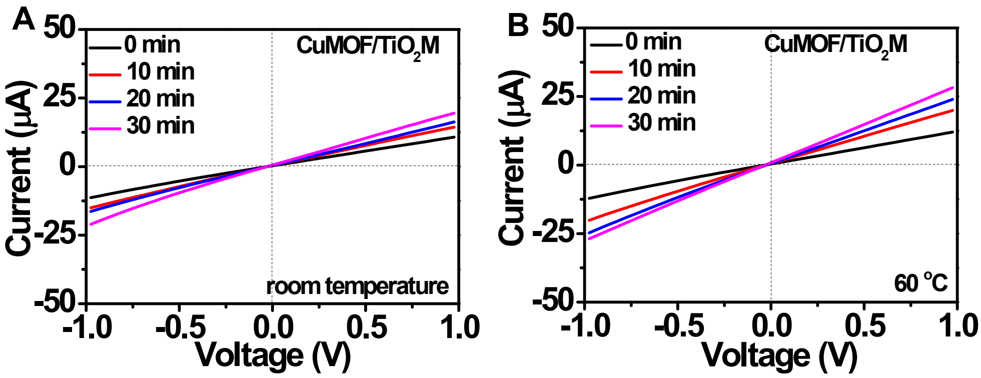

Figure S18. Effect of temperature (A) 25 ºC and (B) 60 ºC on Fenton-like activity of Meso-L-CuMOF through *I*-*V* assay.

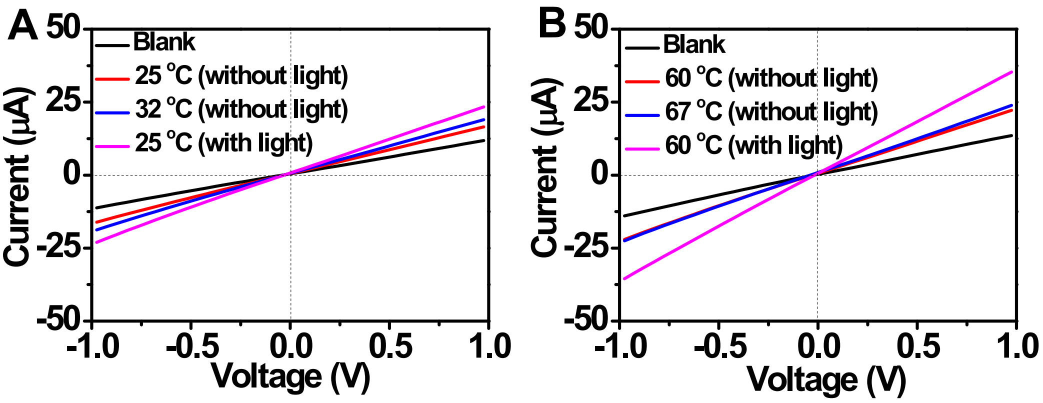

Figure S19. Influence of the temperature and 532 nm-laser irradiation on *I-V* curves of Au@Meso-CuMOF/TiO_2M for 10 μM D-glucose at (A) lower temperatures and (B) higher temperatures.

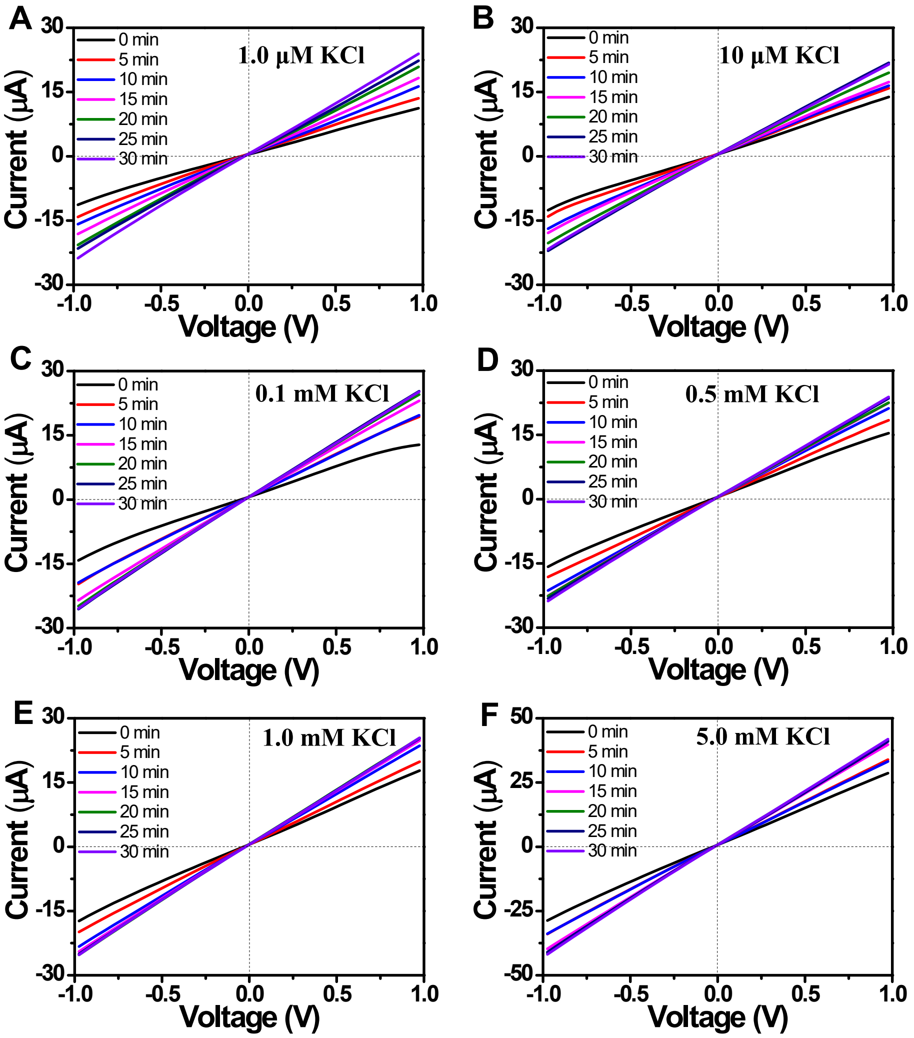

Figure S20. *I-V* properties of Au@Meso-L-CuMOF/TiO_2M in the presence of 10 μM D-glucose in different concentration of KCl (A) 1.0 μM, (B) 10 μM, (C) 0.1 mM, (D) 0.5 mM, (E) 1.0 mM, and (F) 5.0 mM.

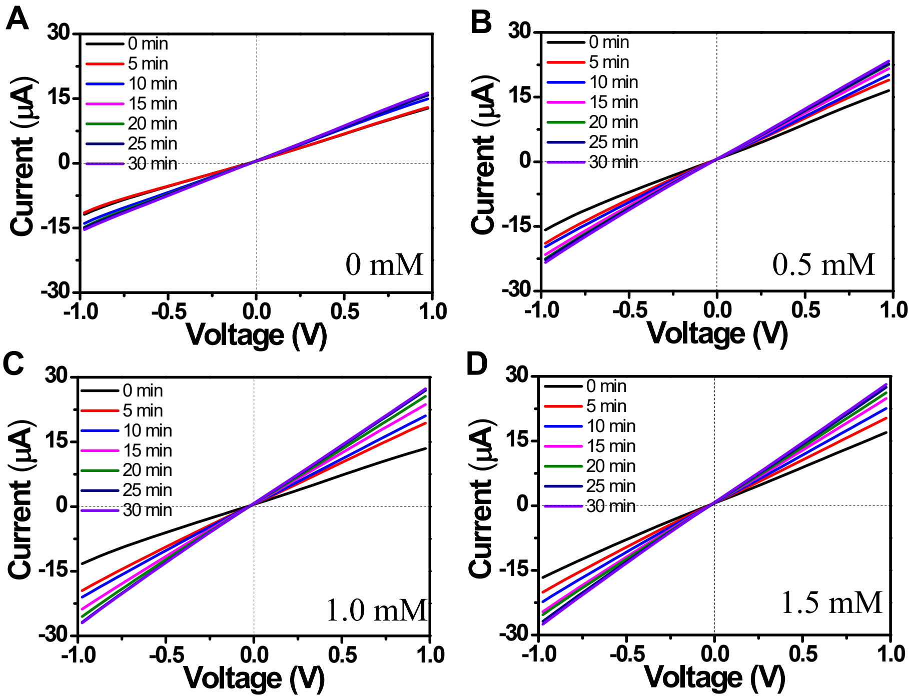

Figure S21. *I-V* properties of Au@Meso-L-CuMOF/TiO_2M in the presence of 10 μM D-glucose under different concentration of ABTS (A) 0 mM, (B) 0.5 mM, (C) 1.0 mM, and (D) 1.5 mM.

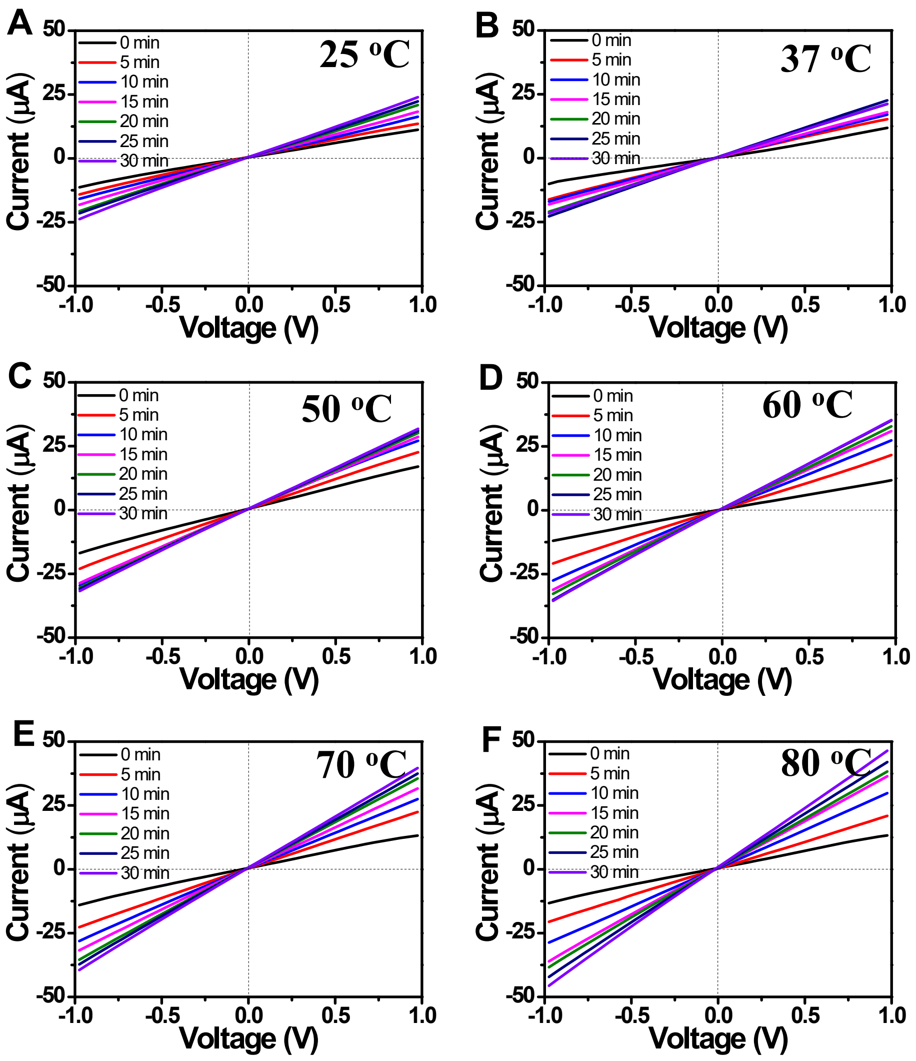

Figure S22. *I-V* properties of Au@Meso-L-CuMOF/TiO_2M in the presence of 10 μM D-glucose at different reaction temperature (A) 25 °C, (B) 37 °C, (C) 50 °C, (D) 60 °C, (E) 70 °C, and (F) 80 °C.

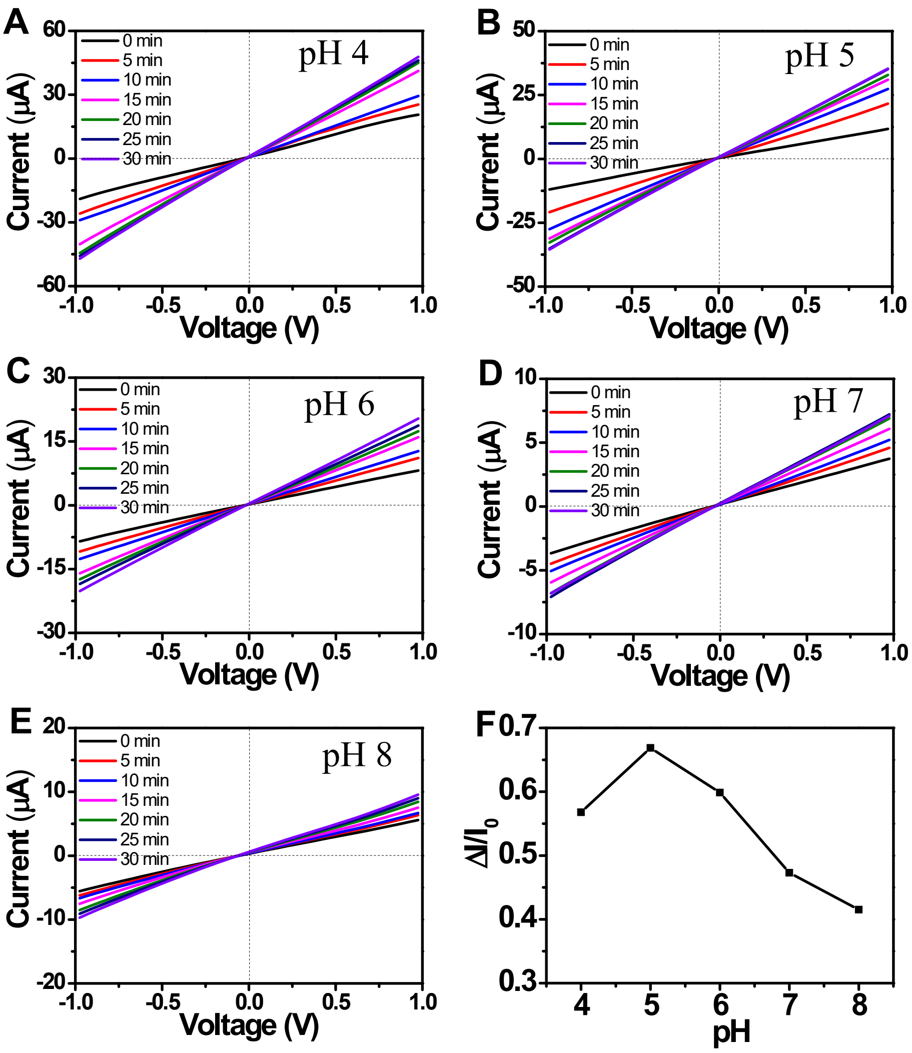

Figure S23. *I-V* properties of Au@Meso-L-CuMOF/TiO_2M in the presence of 10 μM D-glucose at different pH (A) 4 (B) 5 (C) 6 (D) 7, and (E) 8. (F) Ionic current change rate ($\Delta I/I_0$) at +1.0 V in the presence of 10 μM D-glucose at different pH.

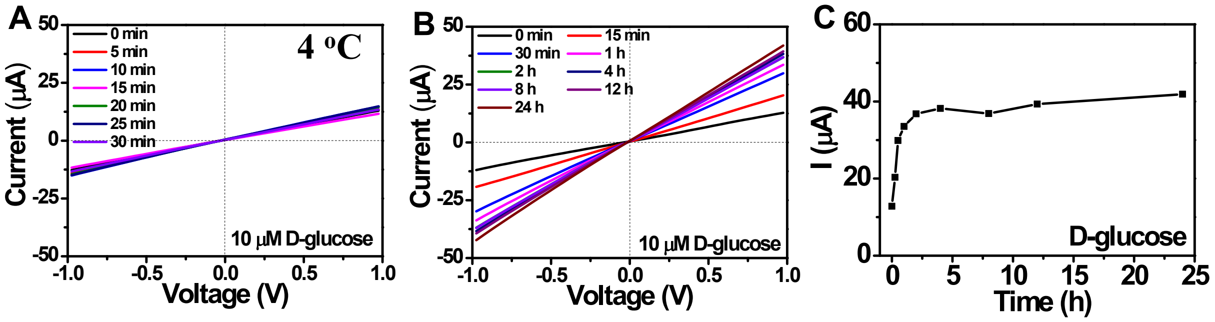

Figure S24. (A) *I-V* properties of Au@Meso-L-CuMOF/TiO_2M in the presence of 10 μM D-glucose at 4 °C. (B) *I-V* properties of Au@Meso-L-CuMOF/TiO_2M for identifying 10 μM D-glucose at a different time at 4 °C. (C) Ionic current at +1.0 V for identifying 10 μM D-glucose with varying times at 4 °C.

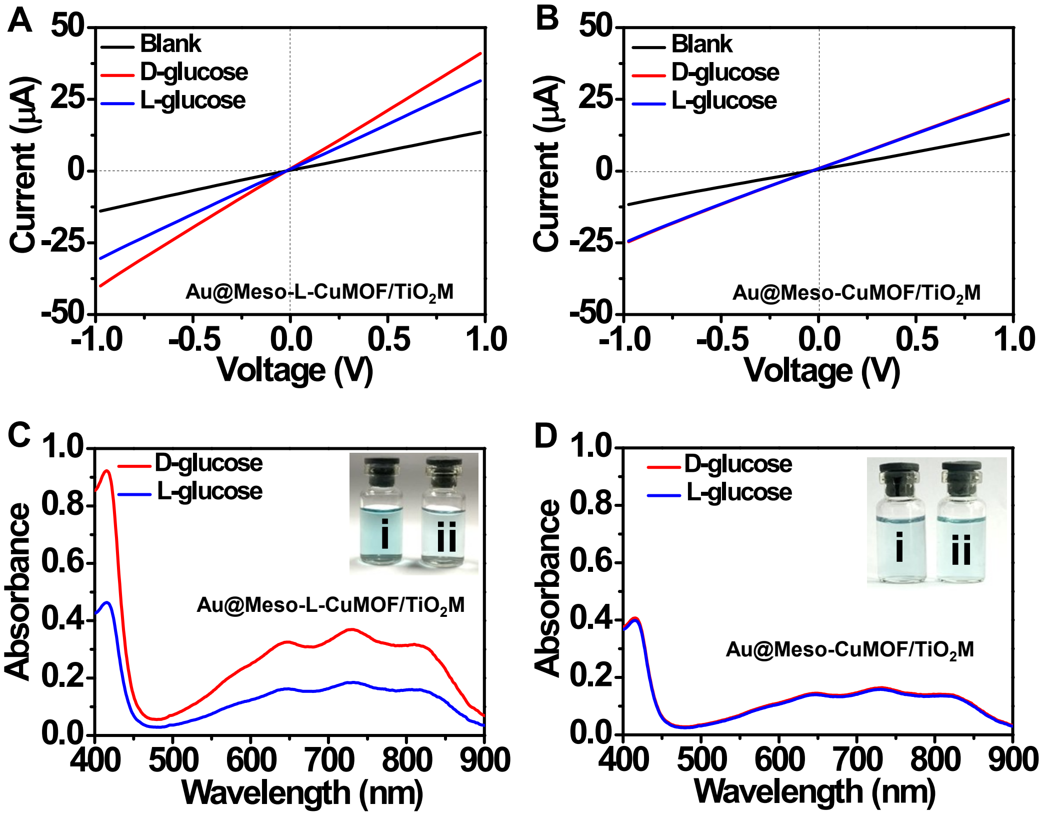

Figure S25. *I-V* curves of (A) Au@Meso-L-CuMOF/TiO_2M and (B) Au@Meso-CuMOF/TiO_2M in the presence of 10 μM L/D-glucose under 532 nm-laser irradiation. UV−vis spectra of (C) Au@Meso-L-CuMOF/TiO_2M and (D) Au@Meso-CuMOF/TiO_2M in 10 μM L/D-glucose and 1.0 mM ABTS. Inset image: the corresponding photographs of solutions corresponding to each curve.

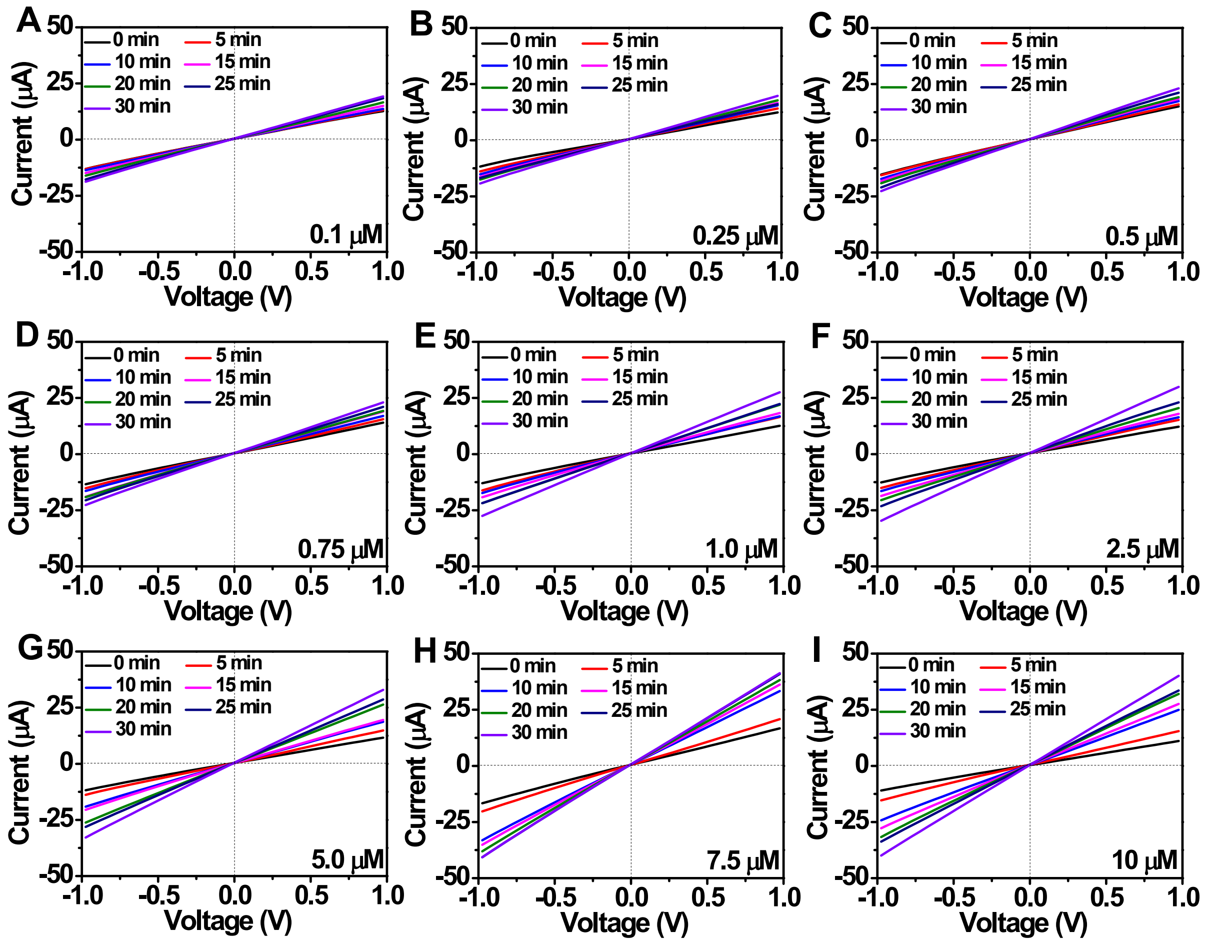

Figure S26. *I–V* curves for sensing different concentrations of D-glucose (A) 0.1 μM, (B) 0.25 μM, (C) 0.5 μM, (D) 0.75 μM, (E) 1.0 μM, (F) 2.5 μM, (G) 5.0 μM, (H) 7.5 μM, and (I) 10 μM. Electrochemical measurements were performed in an aqueous solution containing 1.0 μM KCl, and 1.0 mM ABTS.

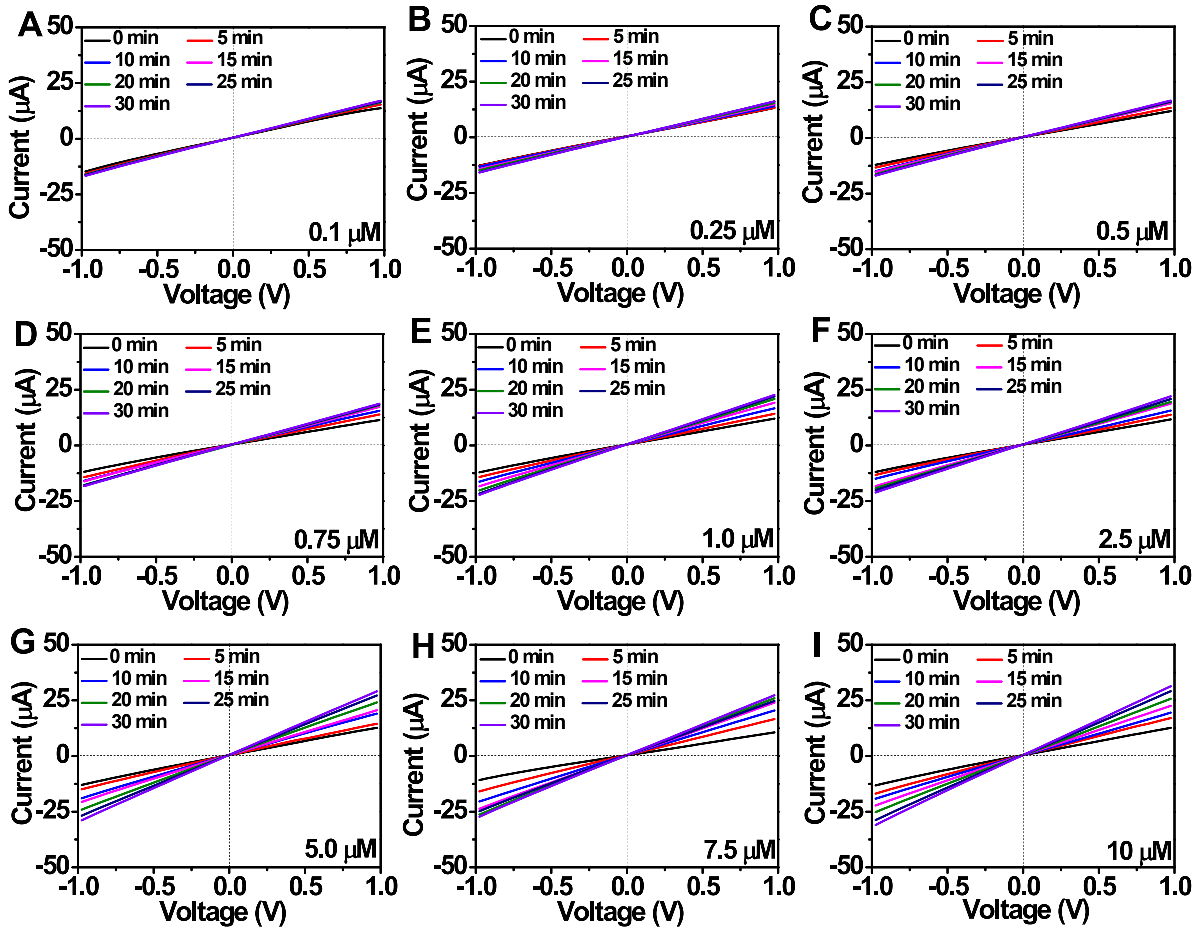

Figure S27. *I–V* curves for sensing different concentrations of L-glucose (A) 0.1 μM, (B) 0.25 μM, (C) 0.5 μM, (D) 0.75 μM, (E) 1.0 μM, (F) 2.5 μM, (G) 5.0 μM, (H) 7.5 μM, and (I) 10 μM. Electrochemical measurements were performed in an aqueous solution containing 1.0 μM KCl, and 1.0 mM ABTS.

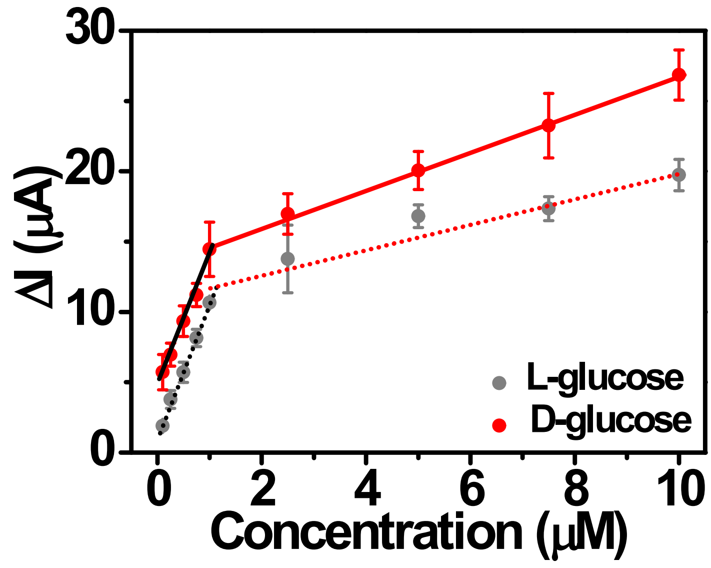

Figure S28. Ionic current changes (ΔI) at +1.0 V and the corresponding calibration plots for different concentrations of L/D-glucose.

In Figure S28, ΔI, defined as $\Delta I = I_0 - I$, where I_0 and I are defined as the ionic current at +1.0 V, derived from I–V curves recorded in an electrolyte containing 1.0 mM ABTS after a 30 min reaction.

Table S3. Comparison of various methods for L/D-glucose detection.

Method	Mechanism	LOD	Reference
Chiral nanochannel-based electrochemical method	The chiral nanochannels modified with a chiral pillar[6]arene-based host-guest system show a high chiral-driven ionic gate for glucose enantiomers	1 mM	[S1]
Gold nanoparticles (AuNPs) with DNA ligands–based colorimetric assay	Random-coiled DNA-capped nanoparticles preferentially catalyze the oxidation of L-glucose, and structured DNA-capped nanoparticles show higher activity toward D-glucose	2 mM	[S2]
Chiral AuNP-based colorimetric strategy	AuNPs modified by polycationic α-cyclodextrin allowed for precise photocontrol recognition catalysis for chiral monosaccharides	100 mM	[S3]
Achiral glucose receptor-based CD assay	Glucose receptor contains bis-ureidobenzenecarboxamido units, binding to D/L-glucose, expected to cause twisting in these chromophores, leading to strong CD signals	40 μM	[S4]
Self-activated cascade reaction based electrochemical assay	AuNPs modified in Meso-L-CuMOF/TiO_2M converts D-glucose (not L-glucose) into gluconic acid and H_2O_2, activating the POD-like activity of PB to trigger the ionic current response further	0.089 μM	This work

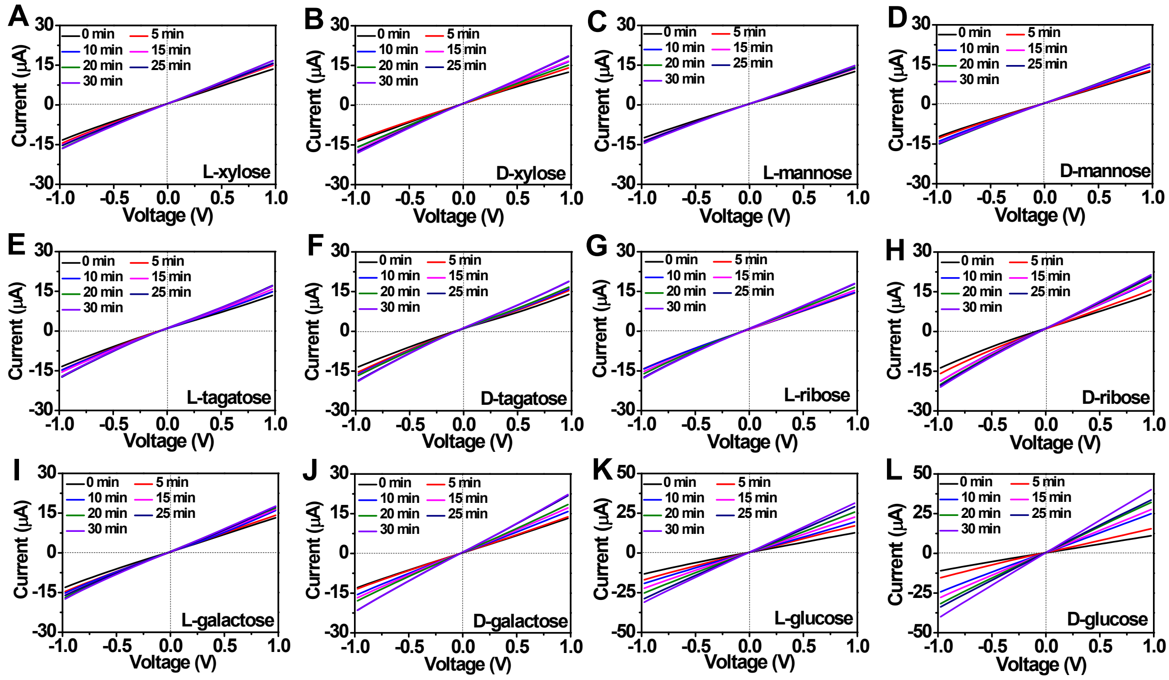

Figure S29. *I–V* curves for sensing different chiral molecules (A) and (B) 10 μM L/D-xylose, (C) and (D) 10 μM L/D-mannose, (E) and (F) 10 μM L/D-tagatose, (G) and (H) 10 μM L/D-ribose, (I) and (J) 10 μM L/D-galactose, (K) and (L) 10 μM L/D-glucose. Electrochemical measurements were performed in an aqueous solution containing 1.0 mM ABTS and1.0 μM KCl solution.

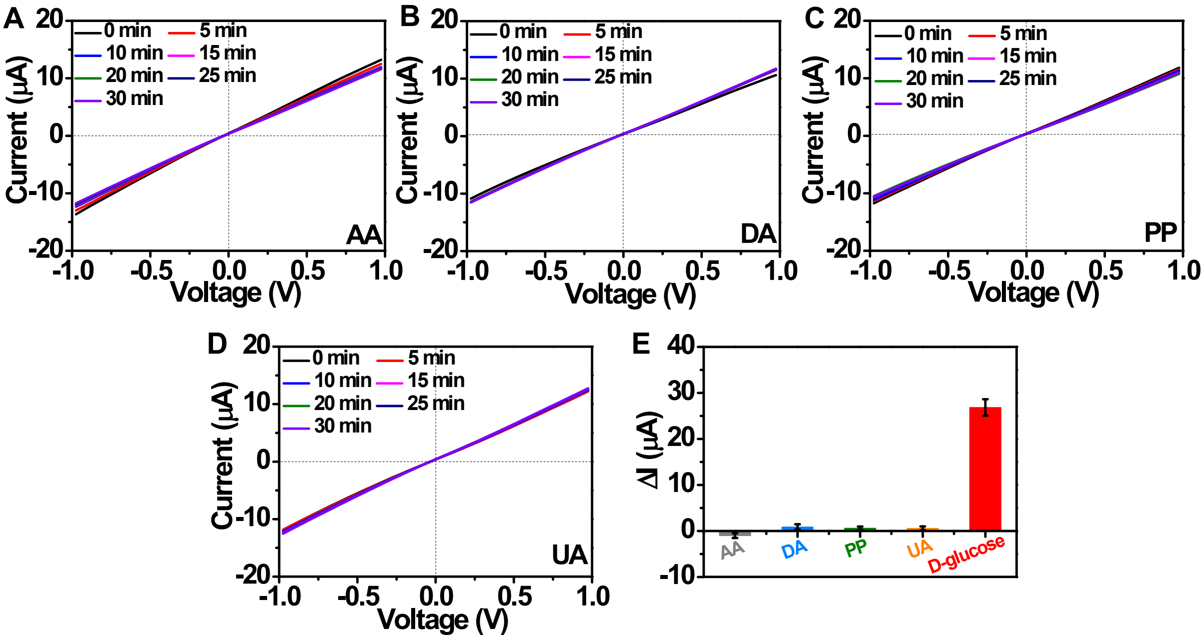

Figure S30. $I-V$ curves for sensing different interfering species (A) 10 μM AA, (B) 10 μM DA, (C) 10 μM UA, (D) 10 μM PP, and (E) ionic current changes (ΔI) at +1.0 V in the presence of different interfering specie. Electrochemical measurements were performed in an aqueous solution containing 1.0 mM ABTS and 1.0 μM KCl solution. The concentration of each interfering species was 10 μM, and the D-glucose concentration was 10 μM.

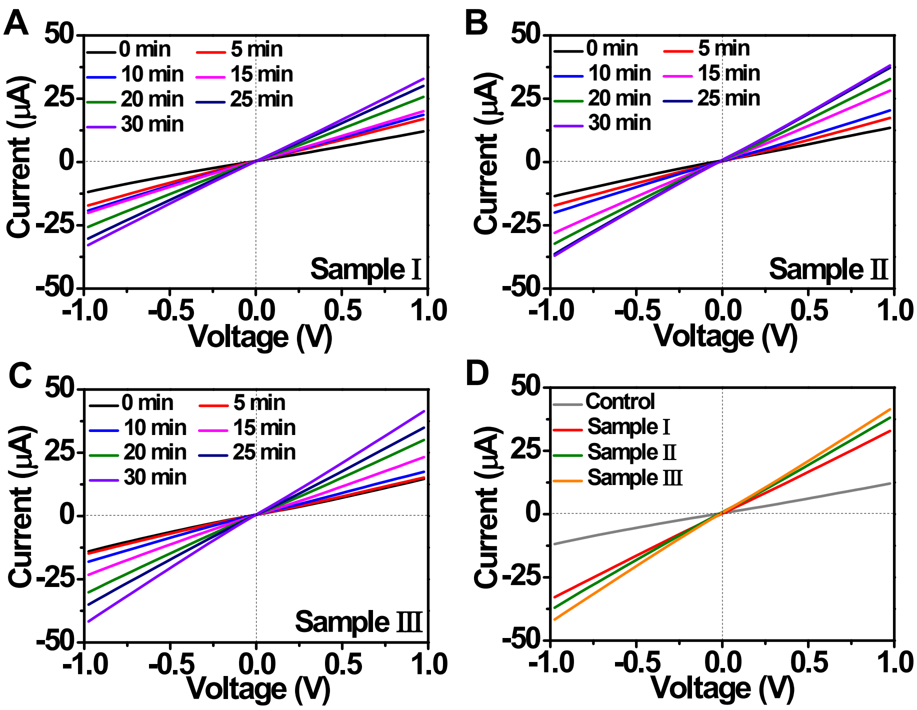

Figure S31. (A-C) $I-V$ curves for sensing D-glucose in serum samples. (D) $I-V$ curves for sensing D-glucose in serum samples after reaction 30 min. In real serum samples, electrochemical measurements were performed in an aqueous solution containing 1.0 mM ABTS and 1.0 μM KCl.

Table S4. Detection of D-glucose in real serum samples using the proposed method.

Sample	Known concentration (mmol L^{-1})	Detected concentration (mmol L^{-1})	Recovery	RSD
Sample I	5.23	5.38 ± 0.11	102.88%	1.89%
Sample II	8.53	8.52 ± 0.23	99.88%	1.05%
Sample III	9.63	9.69 ± 0.34	100.62%	2.36%

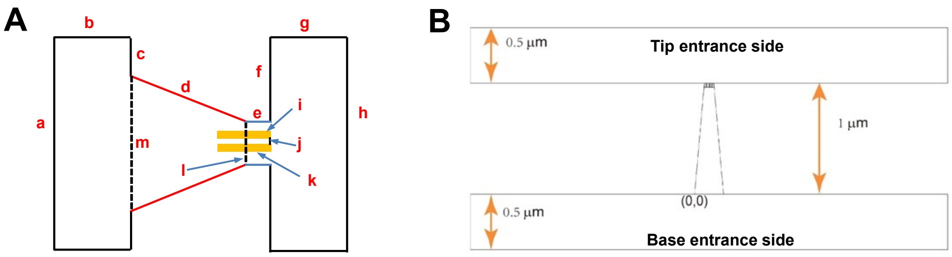

Figure S32. (A) Calculation model of 2D computation domain for heterochannels (Note that the figure is not drawn to scale). (B) Computer domain and boundaries of FEM models.

To calculate the ion distribution, the Comsol Multiphysics 5.5 was used with the "electrostatics (Poisson equation)" and "Nernst-Planck without Electroneutrality" modules. As schematically presented in Figure S25a, the Meso-L-CuMOF nanocrystals are located at the tip-entrance side of TiO_2M. The pore size of Meso-L-CuMOF is ~7.5 nm (Figure 2E), and the spacing between the holes of CuTCA is 0.7 nm.[S5] The thickness of Meso-L-CuMOF is 3.5 µm, based on the SEM image (Figure 1F).

Table S5. Boundary conditions of FEM models.

Boundary	Poisson equation	Nerst-Planck equation
a, h	V_a=applied voltage; V_h=0 V	Concentration
d, l, i, k	Surface charge	Insulation/symmetry
b, c, e, f, g	Zero charge/symmetry	Insulation/symmetry
j, m	Continuity	Insulation/symmetry

*For concentrations of gluconic acid, H_2O_2, and $ABTS^+$, their concentrations are dependent on the catalyzed reaction equation (eq1, eq2, and eq.3)

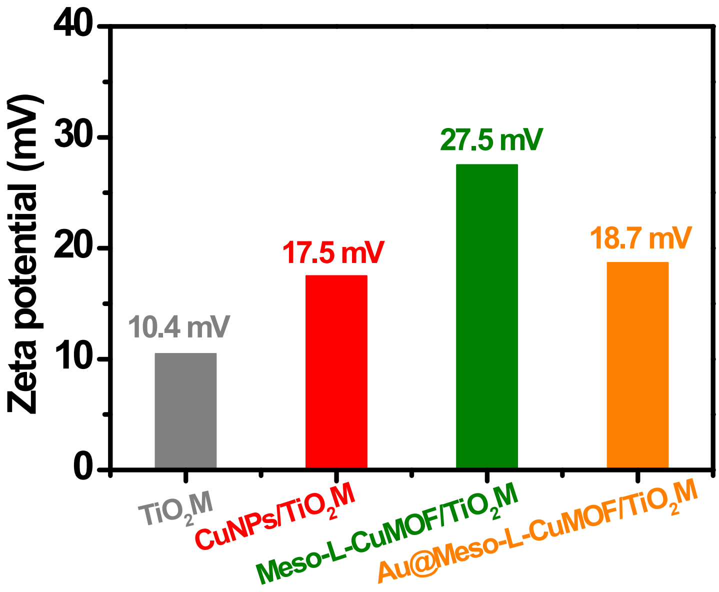

Figure S33. Zeta potential of each step modification in 1.0 μM KCl solution (pH 5).

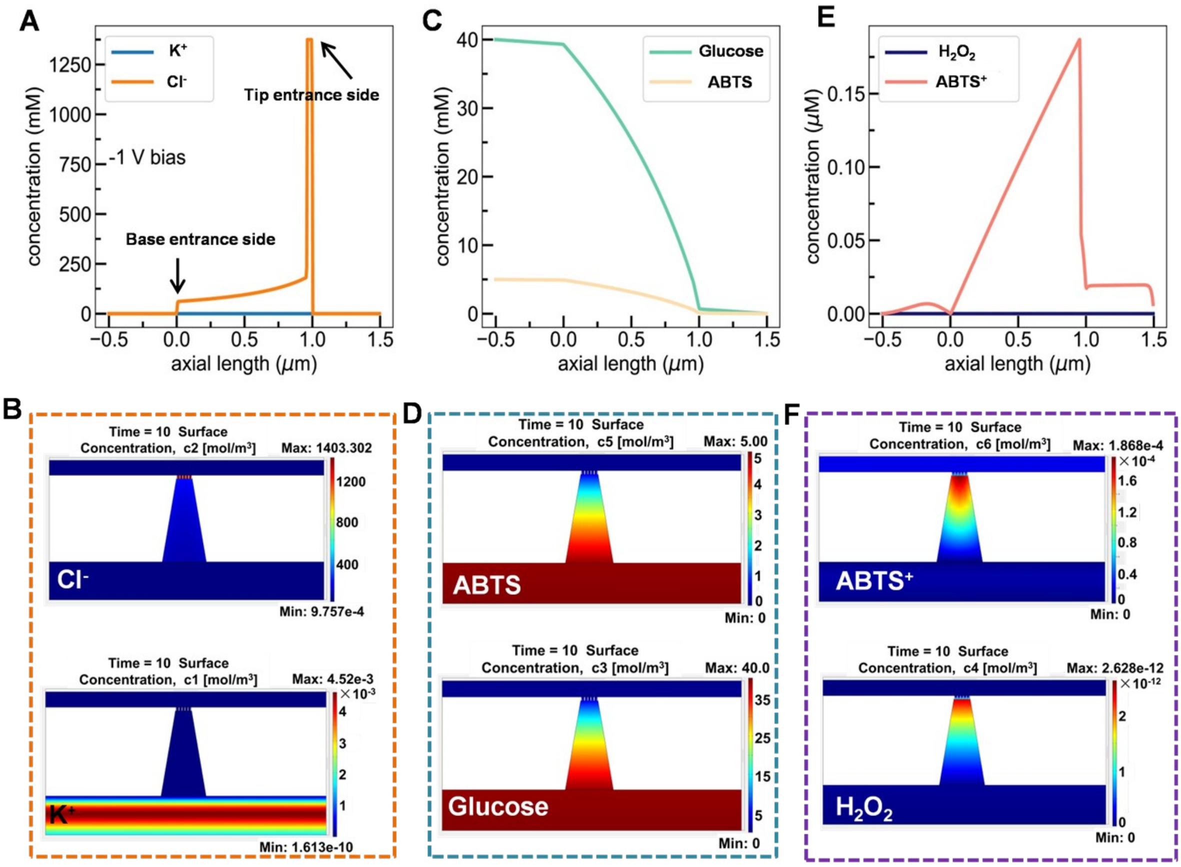

Figure S34. (A) Simulation concentration profiles of KCl and (B) Cl^- and K^+ ions in nanochannel without enzyme catalytic reaction. (C) Simulation concentration profiles of glucose and ABTS along the nanochannel. (D) Simulated ionic concentration images of glucose and ABTS in a nanochannel. (E) Simulation concentration profiles of H_2O_2 and $ABTS^+$ along the nanochannel. (F) Simulated ionic concentration images of H_2O_2 and $ABTS^+$ in a nanochannel. The transmembrane voltage was set as –1.0 V.

References

[1] Y. Sun, F. Zhang, J. Quan, F. Zhu, W. Hong, J. Ma, H. Pang, Y. Sun, D. Tian and H. Li, *Nat.Commun.,* 2018, **9**, 2617.

[2] R. A. Tromans, S. K. Samanta, A. M. Chapman and A. P. Davis, *Chem. Sci.,* 2020, **11**, 3223.

[3] P. Zhan, Z-G. Wang, N. Li and B. Ding, *ACS Catal.,* 2015, **5**, 1489.

[4] L. Chen, Y. Chen, Y. Zhang and Y. Liu, *Angew. Chem. Int. Ed.,* 2021, **60**, 7654.

[5] S. S.-Y. Chui, S. M.-F. Lo, J. P. H. Charmant, G. Orpen and I. D. Williams, *Science,* 1999, **283**, 1148.
